# Spatio-temporal analysis and geostatistical modelling of onchocerciasis prevalence in Nigeria to support elimination efforts

**DOI:** 10.1371/journal.pntd.0014090

**Published:** 2026-03-09

**Authors:** Ayodele Samuel Babalola, Taiwo A. Adekunle, Taiwo P. Babatunde, Yasmeen A. Adeniyi, Omolola Adeniran, Olaitan Omitola, Edore Edwin Ito, Abiodun Olakiigbe, Pam V. Gyang, Emeka Makata, Babatunde Adewale, Olaoluwa P. Akinwale, Olufunmilayo A. Idowu, Olabanji A. Surakat, Adedapo O. Adeogun, Monsuru A. Adeleke

**Affiliations:** 1 Department of Public Health and Epidemiology, Nigerian Institute of Medical Research, Yaba, Lagos, Nigeria; 2 Osun State University, Osogbo, Nigeria; 3 Medical Statistics Programme, Department of Population Health Science, University of Leicester, Leicester, United Kingdom; 4 Neglected Tropical Diseases Control Unit, Federal Ministry of Health, Abuja, Nigeria; 5 Federal University of Agriculture, Abeokuta, Nigeria; 6 Federal University of Medical Sciences, Ila-Orogun, Osun State, Nigeria; 7 Delta State University, Abraka, Nigeria; Universiteit Antwerpen, BELGIUM

## Abstract

Nigeria has made significant progress toward the elimination of onchocerciasis through mass drug administration (MDA) of ivermectin, with ten states recently declared eligible to stop treatment following WHO-recommended epidemiological and entomological assessments. However, reliable spatial prevalence estimates remain necessary to guide elimination strategies, particularly in areas with limited surveillance. We applied model-based geostatistical analysis using Monte Carlo Maximum Likelihood Estimation to assess the spatio-temporal distribution of onchocerciasis prevalence across Nigeria from 1989 to 2024. Climatic, hydrographic, socio-economic, and topographic variables were incorporated to predict prevalence in unsampled locations. Predicted prevalence declined substantially over time. During 1997–2000, 64.9% (24/37) of states had mean predicted prevalence between 10–30%, and 5.4% (2/37) exceeded 30%. By 2017–2020, 70.3% (26/37) of states were classified within the 0–2% category, increasing to 86.5% (32/37) in 2021–2024. Nevertheless, resurgence was observed in selected areas; for example, Taraba State showed an absolute increase of 44.1 percentage points between 2013–2016 and 2021–2024 (p = 0.013). High-prevalence clusters persisted along international and interstate borders, particularly in southern Nigeria. Model performance was strong (correlation between observed and predicted prevalence: 0.80–0.86; RMSE < 0.08). The estimated spatial correlation range increased from 31.93 km (95% CI: 31.92–31.94 km) in 1997–2000 to 180.20 km (95% CI: 180.20–180.20 km) in 2021–2024. Mean annual temperature, rainfall in the driest quarter, elevation, and river flow accumulation were significant predictors of prevalence. These findings underscore the need for complementary approaches such as predictive modelling to strengthen the field surveys in planning and surveillance of the disease. To sustain the progress toward onchocerciasis elimination in Nigeria, there is a need for adaptive, climate-informed strategies, intensified surveillance in high-risk areas, and enhanced coordination, particularly in cross-border and hard-to-reach communities.

## Author summary

Onchocerciasis (river blindness) remains a public health concern in Nigeria, although substantial progress has been made through long-term mass drug administration of ivermectin. Ten states have recently met World Health Organization criteria to stop treatment, but gaps in surveillance data make it difficult to fully understand where transmission may persist. We used advanced spatial modelling techniques to reconstruct changes in onchocerciasis prevalence across Nigeria from 1989 to 2024 and to predict risk in areas without survey data. Our findings show a marked national decline in prevalence over time, with most states now falling within very low prevalence categories. However, localized resurgence was observed in some areas, particularly near international and interstate borders. Environmental factors such as temperature, rainfall, elevation, and proximity to rivers were important predictors of infection risk. These results highlight the value of predictive modelling to complement field surveys and support targeted, climate-informed strategies to sustain elimination efforts.

## Introduction

Onchocerciasis, also known as river blindness, is a neglected tropical disease (NTD) caused by the filarial parasite *Onchocerca volvulus*, which is transmitted to humans through repeated bites by infected blackflies (*Simulium* spp.) has remained a resilient problem to public health efforts [1,2]. The disease causes debilitating symptoms such as intense itching, onchodermatitis, visual impairment, and irreversible blindness [1], with emerging evidence also linking it to epilepsy and nodding syndrome in children [3]. Nigeria bears the heaviest burden globally, accounting for nearly 40% of all cases, making it a critical focus for global elimination efforts [2,4,5]. Over the past two decades, Nigeria has implemented community-directed mass drug administration of ivermectin (MDAi) across various transmission foci, delivering treatment annually or biannually to endemic communities [6]. These efforts have significantly reduced prevalence in many regions [6]. However, persistent transmission remains in several pockets [2,7], particularly in areas with poor access to health services, hard-to-reach or insecure regions, and along cross-border zones where vector breeding conditions are favourable. Furthermore, variations in ecological zones, intervention histories, treatment coverage, and compliance levels contribute to the spatial heterogeneity of disease distribution [8,9].

Despite the scale of Nigeria’s programme, several challenges hinder the goal of onchocerciasis elimination. A major limitation is the lack of comprehensive, high-resolution baseline prevalence maps across the country. Many foci remain unmapped or have outdated and incomplete data, particularly in hypoendemic areas where MDA was historically deprioritized [5]. Additionally, conventional mapping strategies—such as those used during the Rapid Epidemiological Mapping of Onchocerciasis (REMO)—relied heavily on nodule palpation and were primarily designed to guide treatment decisions [10], not elimination planning. These methods often fail to capture spatial heterogeneity or the true spatial scale of transmission. Moreover, areas with unknown onchocerciasis endemicity may pose a threat to elimination efforts, as they could serve as sources of re-introduction in regions where transmission has been interrupted [11].

Understanding the fine-scale spatial variation in infection risk is therefore critical for achieving elimination—particularly in Nigeria, a geographically vast and ecologically diverse country. Blackfly breeding habitats are concentrated in specific river basins with suitable environmental conditions, and local socio-demographic factors influence both exposure and compliance with treatment [2,4]. Accurate mapping of transmission risk including the spatial scale of transmission—is essential for identifying residual hotspots, prioritizing surveillance, provide current information on endemicity, and optimizing the allocation of limited resources.

Spatio-temporal geostatistical modelling offers a powerful tool to address these challenges. By leveraging point prevalence data and integrating relevant environmental, climatic, and demographic covariates, these models generate continuous risk surfaces across time and space. Such maps enable the extrapolation of prevalence to unsampled areas, account for spatial autocorrelation, and quantify the spatial scale of transmission, thereby supporting evidence-based decisions for planning interventions and evaluating their impact [12,13]. Importantly, this approach shifts the focus from administrative boundaries to epidemiologically meaningful spatial units, enabling more targeted and cost-effective strategies for surveillance and treatment.

In this study, we present a comprehensive spatio-temporal geostatistical analysis of *O. volvulus* prevalence in Nigeria, using survey data collected between 1989 and 2024. We integrate data from the Expanded Special Project for the Elimination of Neglected Tropical Diseases (ESPEN) [14] and peer-reviewed literature, alongside covariates representing ecological suitability and population vulnerability. Our objectives are to reconstruct the historical distribution of onchocerciasis, identify potential foci of persistent transmission, assess the spatial scale of transmission, and support Nigeria’s national elimination programme in its final push towards transmission interruption and elimination. Ultimately, our results are intended to guide the more strategic deployment of MDA, enhance the targeting of surveillance, and strengthen cross-border coordination where necessary to prevent recrudescence.

## Materials and methods

### Study area and prevalence data

**Fig 1:**
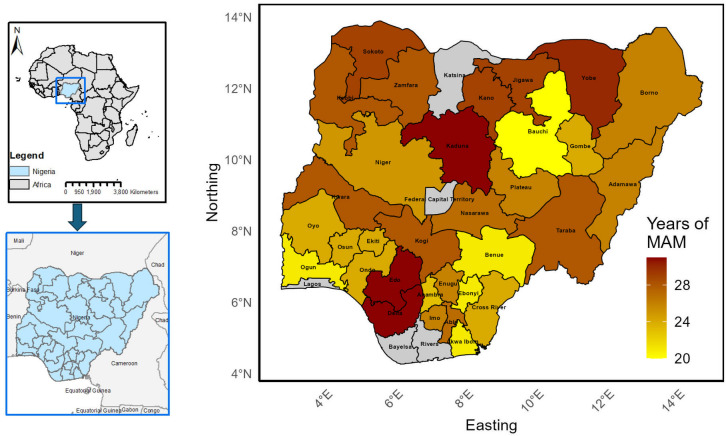
Duration of MAM rounds in different States (implementation unit) across Nigeria. This figure was created by the authors in R programming software (R version 4.1.2, Vienna, Austria). Available at https://www.R-project.org/. The Nigerian shapefile was obtained from World Bank Data Catalog (https://data.humdata.org/dataset/geoboundaries-admin-boundaries-for-nigeria), an Open license standardized resource of boundaries (i.e., state, county) for every country in the world.

**Fig 2:**
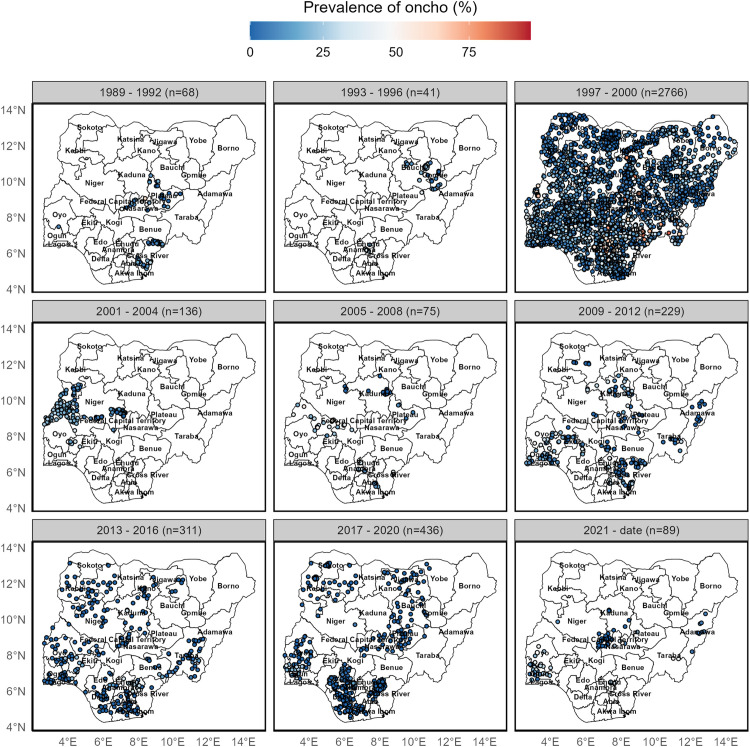
Reported prevalence of onchocerciasis at community level in Nigeria between 1989 to 2024. This figure was created by the authors in R programming software (R version 4.1.2, Vienna, Austria). Available at https://www.R-project.org/. The Nigerian shapefile was obtained from World Bank Data Catalog (https://data.humdata.org/dataset/geoboundaries-admin-boundaries-for-nigeria), an Open license standardized resource of boundaries (i.e., state, county) for every country in the world.

Data on the prevalence of *Onchocerca volvulus* infection, including specific geographic coordinates for Nigeria, were obtained from the publicly available Expanded Special Project for Elimination of Neglected Tropical Diseases (ESPEN) database (ESPEN, 2020) and from peer-reviewed scientific literature published between 1989–2024. To capture spatio-temporal trends in prevalence and surveillance coverage, we grouped the data into distinct time periods (see Fig 2). The summary of the empirical survey characteristics and prevalence statistics by survey period, including number of sites, sample size, spatial dispersion, and state coverage are presented in S1 File. Nigeria comprises 36 states and the Federal Capital Territory (FCT) (Fig 1), with an estimated population exceeding 200 million [15]. The country generally has a tropical climate characterized by two main seasons: a rainy season (May to October) and a dry season (November to April). Mean annual weather conditions range from 24.0 °C to 30.2 °C (temperature), 31.1% to 85% (relative humidity), and 314 mm to 1,871 mm (rainfall) [16]. Vegetation is predominantly Guinea, Sudan, and Sahel savannah in the northern States, while the southern states feature mangrove forests, tropical rainforests, and parts of the Guinea savannah [16]. Mass Administration of Medicines (MAM) has been implemented for at least 20 years in most parts of the country, with the southern states generally initiating treatment later than their north (Fig 1).

### Environmental, socio-demographic, and climatic predictors

A total of 41 covariates relevant to *O. volvulus* infection prevalence and *Simulium* ecology, based on published literature, were obtained from various sources and exported as raster layers at a 1 km resolution (Table 1). Raster layers with higher resolutions were down sampled using mean aggregation to 1 km resolution, creating a raster stack of uniform scale [11]. The raster data were primarily processed using the GDAL library (GDAL/OGR Geospatial Data Abstraction Library, Open Source Geospatial Foundation, https://gdal.org/) in a Linux Bash environment, with additional processing performed in R [25,26]. All rasters were reprojected to the World Geodetic System 1984 (WGS84) coordinate reference system. The covariates were then cropped to the boundary of Nigeria, and a raster stack was prepared. Finally, values of the covariates at each sample location were extracted from the raster stack.

**Table 1:** Data sources and properties of environmental, Socio-economic and climatic covariates explored to model the prevalence of Onchocerciasis in Nigeria.

Variable type	Database name	Variable description	Spatial resolution	Number of layers selected	Source
Geomorphology	Geomorpho90m	Elevation, Hill shade, Aspect, Slope	3 arc second	4	[17]
Hydrology	Hydrography90m	Accumulation, Stream power index, distance to the nearest stream/river, compound topographic index	3 arc second	4	[17]
Climate	CHELSA	Bioclimatic (bio 1–19)	30 arc second	19	[18]
Climate	Ecoregions2017	Ecoregions	30 arc second	1	[19]
Population	GHS	human population	100 meter	1	[20]
Earth cover	ESA WorldCover	land cover	10 meter	1	[21]
Earth cover	Sentinel mosaic	NDVI, NDTI, NDWI	10 meter	3	[20]
Livestock	GLW3	livestock density (Pig, goats, cattle, horse, donkey, chicken	30 arc second	6	[22]
Water	FLO1K	flow discharge	30 arc second	1	[23]
Water	GSW	surface water occurrence and occurrence proximity	30 arc second	1	[24]

### Variable selection procedures for the geostatistical model

To select the optimal set of covariates for the geostatistical model, firstly, pairwise correlations was assessed using Pearson’s correlation coefficient (Figs A-E in S1 Appendix) Covariate pairs with high correlation values (Pearson’s r > 0.7) were identified, and only one variable from each correlated pair was retained for modelling [27]. The retained variable was selected after assessing the strength and clarity of the relationship with response variable with a scatterplot (Figs A-I and Table A in S2 Appendix) [27]. Next, both forward and backward stepwise selection was applied to identify a parsimonious set of covariates for predicting the prevalence of onchocerciasis. This was done by fitting a non-spatial generalized linear model (GLM) relating the prevalence of onchocerciasis to the candidate covariates. The final set of covariates was chosen based on the model with the lowest Akaike Information Criterion (AIC), beyond which the inclusion of any additional covariates did not improve model performance.

### Model based geostatistical analysis

The binomial distribution, which is the suitable distribution for dichotomous observations, was adopted for analysing the onchocerciasis prevalence data, with *n* representing the number of trials at each location. Before fitting a binomial geostatistical model, we assessed whether the residual variation in the response variable—unexplained by the covariates—exhibited spatial correlation. This was done by first fitting a generalized linear model (GLM) using the selected covariates and extracting the residuals at each sampling location. The residuals from the GLM were then used to compute the empirical variogram, following the method described by [13] (Fige A in S3 Appendix). The variogram was employed to visually assess the presence of spatial correlation in the residual variation.

Subsequently, a binomial geostatistical model was fitted to the logit-transformed onchocerciasis prevalence, *p(xk)*, at each location *xk* in the dataset. Let *yk* represent the observed prevalence of onchocerciasis at location *xk*. Then, the sampling distribution of *yk* follows a binomial distribution with probability *p*. The variation in *p(x)* was modeled as a function of environmental predictors δ *(x)* and a spatially structured random effect *S(x)* along with an unstructured random effect *Z*. The geostatistical model is given as:


$$\text{log}\left\{\frac{p\left(x_{i}\right)}{\text{1}\;-\;p\left(x_{i}\right)}\right\}\;=\;\sum j\;\delta_{j}\left(x_{i}\right)\beta_{j}\;+\;S\left(x_{i}\right)\;+\;Z_{i}$$

This modelling approach have been previously described in greater detail [12,13]. In the model, $\;\delta_{j}\left(x_{i}\right)$ r represents the cumulative effect of the covariates, while $\beta_{j}$ denotes the coefficients of the explanatory variables. The term $S(x_{i},+\;Z_{i}$ captures the sum of spatial and unstructured random variations in $p\left(x_{i}\right)$ that are not explained by the covariates. The parameters of the binomial geostatistical model were estimated using the Monte Carlo Maximum Likelihood (MCML) method implemented in the PrevMap package in R. The empirical variogram provided estimates of the spatial correlation structure (σ², ∅), which informed the MCML simulations. It was assumed that the Gaussian process $S\left(x_{i}\right)$ follows an exponential covariance function with kappa, *k* = 0.5.

A spatially continuous predictive surface of onchocerciasis point prevalence was generated using a 1 km × 1 km resolution prediction grid covering the boundaries of Nigeria. The predicted prevalence at each grid location was defined as the mean of the location-specific predictive distribution. The 5th and 95th percentiles of this distribution were used to assess the precision of the predictions (Fig B in S3 Appendix).

### Model validation

We validated our geostatistical model by assessing its predictive performance using 5-fold cross-validation. All survey data were randomly split into five groups. Each group was sequentially held out while the model was fitted to the remaining four groups. The predictive performance was then evaluated on the held-out group. The withheld data were compared with the model predictions to summarize performance using correlation (between the in-sample observed data and the predicted outcome) and root mean square error (RMSE) [27].

## Results

### Data summaries

The reported community-level prevalence of onchocerciasis in Nigeria from 1989 to 2024 is presented in Fig 2. To effectively capture the spatio-temporal patterns in relation to surveillance efforts and prevalence, the reports were grouped by four-year intervals. The results show that most surveillance reports emerged from 1997–2000. The periods between 2017–2020, 2013–2016, and 2009–2012 also witnessed a high number of reports in descending order (Table A in S4 Appendix). Datasets from these periods are spatially distributed well enough to capture geographic patterns and variations in prevalence, enabling geostatistical modelling of the spatio-temporal trends of onchocerciasis in the country. Additionally, the results showed reported prevalence ranging from 0% to 85% across the country, with prevalence generally declining over time (Fig 2).

### Estimated empirical mean prevalence at different administrative levels from 1989 to the present

**Fig 3:**
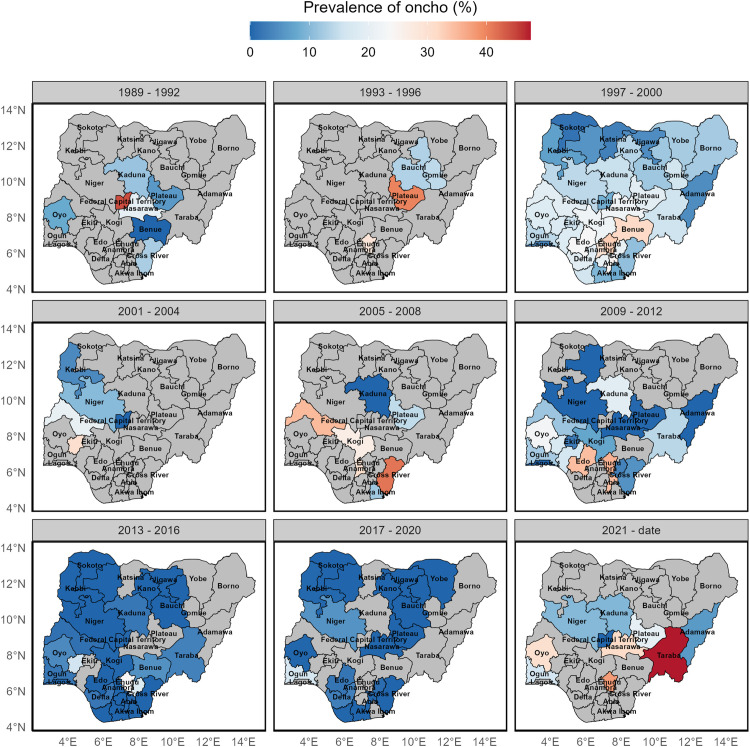
Reported prevalence of onchocerciasis at state level (transmission zones) in Nigeria between 1989 to 2024. States in grey connotes missing data. This figure was created by the authors in R programming software (R version 4.1.2, Vienna, Austria). Available at https://www.R-project.org/. The Nigerian shapefile was obtained from World Bank Data Catalog (https://data.humdata.org/dataset/geoboundaries-admin-boundaries-for-nigeria), an Open license standardized resource of boundaries (i.e., state, county) for every country in the world.

The mean empirical prevalence at the state level (transmission zones as defined in Nigeria context by the Federal Ministry of Health), ranges from 0–50% (Fig 3). Mean prevalence was generally higher across most transmission zones during the 1997–2000 period. In subsequent years, some transmission zones, such as those in the northern parts of the country

had lower mean onchocerciasis prevalence. Others in the southern and north-central areas (such as Kwara, Kogi, Anambra, Cross River, Edo, Enugu, and Abia States) reported higher prevalences compared to the baseline period (1989–2000).

By the 2013–2016 period, mean onchocerciasis prevalence had declined to very low levels in most States (Table B in S4 Appendix), with estimates generally below 1% (range: 0.00–0.37%), and 95% confidence intervals that typically overlapped zero (e.g., Abia: 0.37%, 95% CI: −0.36–1.10; Akwa Ibom: 0.10%, 95% CI: −0.03–0.23; Federal Capital Territory: 0.29%, 95% CI: −0.28–0.86). In contrast, several States recorded appreciably higher prevalences during this period, notably Osun (15.93%, 95% CI: 10.36–21.51), Ebonyi (18.26%, 95% CI: 9.86–26.66), Enugu (23.66%, 95% CI: 14.63–32.68), and Benue (5.17%, 95% CI: −0.37–10.72), indicating persistent transmission in these areas (Fig 3). However, in the most recent period (2021–2024), mean prevalence increased in several States that had previously recorded very low prevalences, suggesting either a resurgence of transmission or improved detection in certain locations, although estimates for this period should be interpreted cautiously due to more limited data availability (Fig 3).

**Fig 4:**
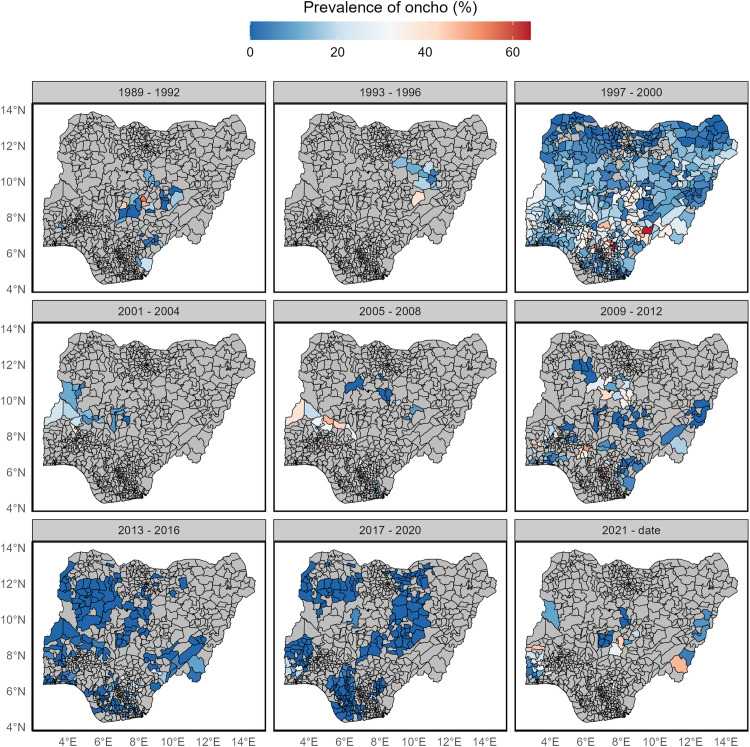
Reported prevalence of onchocerciasis at LGA level (Sub-units) in Nigeria between 1989 to 2024. LGAs in grey connotes missing data. This figure was created by the authors in R programming software (R version 4.1.2, Vienna, Austria). Available at https://www.R-project.org/. The Nigerian shapefile was obtained from World Bank Data Catalog (https://data.humdata.org/dataset/geoboundaries-admin-boundaries-for-nigeria), an Open license standardized resource of boundaries (i.e., state, county) for every country in the world.

Mean prevalence estimated at the local government area (LGA) level (Fig 4) show a similar pattern over the years, with prevalences ranging from 0–60% across all time. The results also highlighted a few pockets of LGAs in different states where prevalences above 30% were reported from 2021 to 2024 (Fig 4).

**Fig 5:**
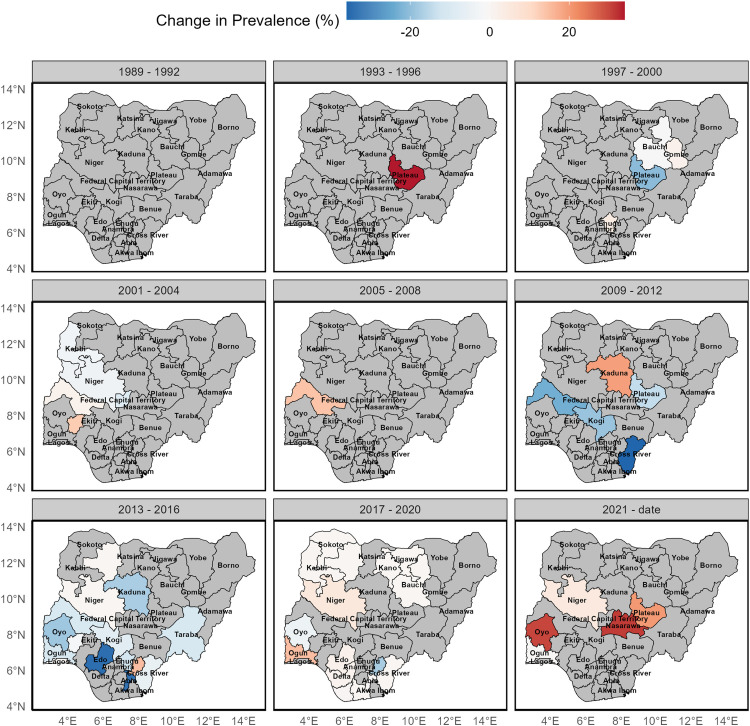
Change in prevalence of onchocerciasis in Nigeria over the period of 30 years. States in grey connotes missing data. This figure was created by the authors in R programming software (R version 4.1.2, Vienna, Austria). Available at https://www.R-project.org/. The Nigerian shapefile was obtained from World Bank Data Catalog (https://data.humdata.org/dataset/geoboundaries-admin-boundaries-for-nigeria), an Open license standardized resource of boundaries (i.e., state, county) for every country in the world.

Fig 5 summarizes the absolute percentage change in observed onchocerciasis prevalence at the state level relative to the preceding period, providing a direct measure of effect size (Table C in S4 Appendix). Large reductions in prevalence (often exceeding 20 percentage points) were observed in several states. For instance, Anambra State experienced a reduction of 30.8 percentage points between 2005–2008 and 2013–2016 (p < 0.0001, Table C in S4 Appendix), while Cross River State showed a decline of 35.9 percentage points between 2005–2008 and 2009–2012 (p = 0.05). Conversely, large increases were observed in later periods, including Plateau State, which recorded an increase of 33.9 percentage points during the 1993–1996 period, and Taraba State, which increased by 44.1 percentage points between 2013–2016 and 2021–2024 (p = 0.013).

In the most recent period (2017–2024), the available data indicate substantial increases in prevalence in several states, with some exhibiting large absolute changes despite wide confidence intervals (Table C in S4 Appendix). However, statistical inference was not possible for some state–period comparisons due to limited data availability, resulting in undefined confidence intervals or p-values (Table C in S4 Appendix). These findings should therefore be interpreted with caution, although the magnitude and direction of observed changes highlight important spatial and temporal variation in onchocerciasis prevalence across Nigeria (Fig 5).

### Predictive Model-Based Geostatistical Risk Maps of Onchocerciasis Prevalence in Nigeria

**Fig 6:**
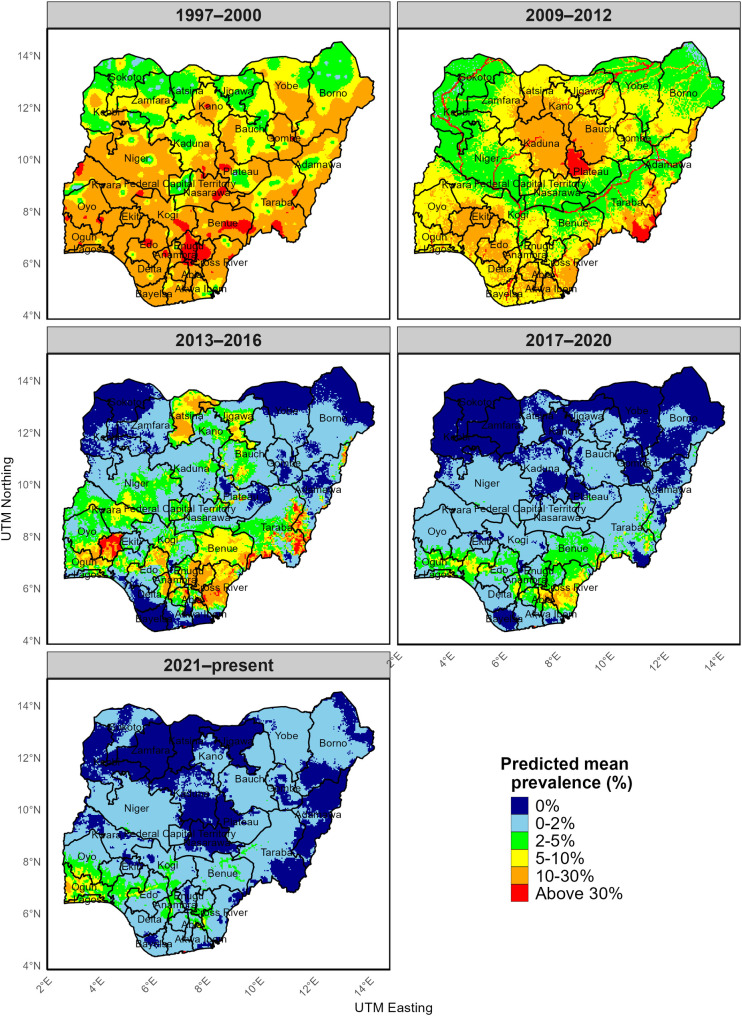
Map showing predicted spatio-temporal prevalence risk of onchocerciasis in Nigeria at specific location level. This figure was created by the authors in R programming software (R version 4.1.2, Vienna, Austria). Available at https://www.R-project.org/. The Nigerian shapefile was obtained from World Bank Data Catalog (https://data.humdata.org/dataset/geoboundaries-admin-boundaries-for-nigeria), an Open license standardized resource of boundaries (i.e., state, county) for every country in the world.

**Fig 7:**
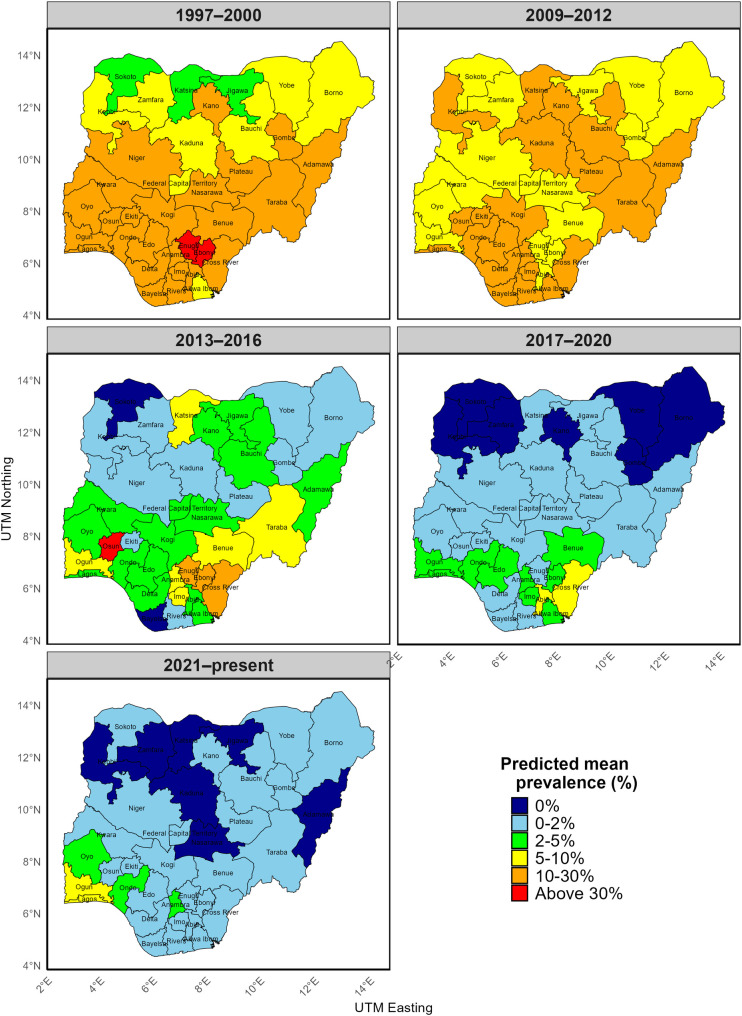
Map showing classified predicted spatio-temporal prevalence risk of onchocerciasis in Nigeria at State (Transmission Zones) level. This figure was created by the authors in R programming software (R version 4.1.2, Vienna, Austria). Available at https://www.R-project.org/. The Nigerian shapefile was obtained from World Bank Data Catalog (https://data.humdata.org/dataset/geoboundaries-admin-boundaries-for-nigeria), an Open license standardized resource of boundaries (i.e., state, county) for every country in the world.

Figs 6 and 7 present the predicted spatio-temporal prevalence of onchocerciasis at the community and State (transmission zone) levels. During the 1997–2000 period, predicted mean prevalence was high across much of Nigeria, with 24 of 37 states (64.9%) classified in the 10–30% category and 2 of 37 states (5.4%) exceeding 30% (Table D in S4 Appendix). High predicted prevalence was particularly widespread in southern Nigeria during this period, where 14 of 17 states (82.4%) were classified in the 10–30% category and 2 of 17 states (11.8%) exceeded 30% (Table E in S4 Appendix).

By 2009–2012, predicted onchocerciasis prevalence in the northern and north-central regions declined from approximately 10–30% to 2–5%, with a consistent nationwide reduction over time ($F_{\text{4},\text{1}\text{4}\text{4}}$ = 91.17, p < 0.001; Tables F-H in S4 Appendix). In 2017–2020 and 2021–present, areas with predicted prevalence ≥10% were limited to a few locations. During 2013–2016, higher predicted prevalence was concentrated along several state and international borders, including Oyo–Ogun, Enugu–Ebonyi, and Adamawa–Cameroon/Taraba. In the most recent period, relatively higher prevalence persisted mainly around border areas such as Ogun–Benin Republic, Oyo–Ogun, Ondo–Edo, and Abia–Cross River (Fig 6).

At the state level, no state had a dominant predicted prevalence ≥10% in either 2017–2020 or 2021–present. In 2017–2020, 70.3% (26/37) of states were classified in the 0–2% category, increasing to 86.5% (32/37) in 2021–present (Fig 6). In these periods, locations with predicted prevalence ≥10% were restricted to small, localized clusters, mainly along riverine and border zones. In contrast, during 2013–2016, predicted prevalence ≥10% covered larger areas within several states. Although only four states (10.8%) had state-level mean predicted prevalence ≥10%, these states contained widespread areas exceeding the 10% threshold rather than isolated foci.

Similar trends are evident at the transmission-zone (state) level (Fig 7), where many states moved from predicted prevalence of 5–10% in 2009–2012–0–2% by 2017–2020. However, relatively higher predicted prevalence persisted in parts of the southern region in recent years, with Ogun and Lagos State remaining in the 5–10% category compared with other states (Fig 7).

### Parameter estimates of the final geostatistical models for spatio-temporal predictions of onchocerciasis in Nigeria

Table 2 presents the parameter estimates of the final geostatistical models predicting onchocerciasis prevalence across five time periods in Nigeria. Overall, the in-sample observed data, and the model predictions showed a correlation coefficient ranging from 0.80 to 0.86 across all time periods, with a root mean square error (RMSE) of less than 0.08. In the period between 1997–2000, a negative association was observed between latitude and prevalence (β = -0.0021, 95% CI: -0.0096 to 0.0055). The estimated spatial correlation range was 31.93 km (95% CI: 31.92 to 31.94 km).

**Table 2:** Estimates (median; 95% confidence interval) of the final geostatistical models for spatio-temporal predictions of onchocerciasis in Nigeria.

Period (year)	Predictor	Estimate (95% CI)
1997–2000	Intercept	-0.8573 (-2.2391, 0.5244)
	Lattitude	-0.0021 (-0.0096, 0.0055)
	Spatial variance	1.2805 (1.1527, 1.4083)
	Spatial scale (km)	31.9295 (31.9211, 31.9379)
2009–2012	Intercept	-3.0804 (-4.1213, -2.0395)
	accumulation	14.7313 (10.1432, 19.3193)
	Annual mean temperature	-0.9054 (-1.4995, -0.3113)
	Spatial variance	5.9446 (5.8897, 5.9995)
	Spatial scale (km)	64.2745 (64.2686, 64.2804)
2013–2016	Intercept	-5.6458 (-6.2491, -5.0426)
	Annual mean temperature	-0.9692 (-1.658, -0.2804)
	Livestock (goat)	0.3947 (0.2439, 0.5456)
	Slope	0.3109 (0.2028, 0.4189)
	Rainfall (driest quarter)	-0.6603 (-1.1463, -0.1742)
	Elevation	-0.9278 (-1.5924, -0.2632)
	Spatial variance	1.7123 (1.5322, 1.8924)
	Spatial scale (km)	97.117 (97.1133, 97.1207)
2017–2020	Intercept	-5.5115 (-6.3703, -4.6527)
	Annual mean temperature	-1.3122 (-1.8166, -0.8078)
	Livestock (donkey)	-0.1924 (-0.3072, -0.0775)
	Slope	0.1052 (0.0339, 0.1764)
	Rainfall (driest quarter)	-0.9068 (-1.2871, -0.5266)
	Elevation	-1.5369 (-2.0252, -1.0485)
	Spatial variance	1.5563 (1.328, 1.7846)
	Spatial scale (km)	168.6401 (168.6377, 168.6425)
2021–2024	Intercept	-6.234 (-7.3241, -5.1438)
	Annual mean temperature	-1.5412 (-2.1546, -0.9277)
	Annual rainfall	1.0073 (0.4107, 1.604)
	Livestock (cattle)	0.7432 (0.5806, 0.9057)
	Rainfall (driest quarter)	-1.7723 (-2.5141, -1.0305)
	Elevation	-1.7786 (-2.4162, -1.1411)
	Spatial variance	2.1819 (2.0245, 2.3393)
	Spatial scale (km)	180.2016 (180.1996, 180.2036)

In 2009–2012, accumulation was positively associated with prevalence (β = 14.73, 95% CI: 10.14 to 19.32), while annual mean temperature was negatively associated (β = -0.91, 95% CI: -1.50 to -0.31). The spatial correlation range was 64.27 km (95% CI: 64.27 to 64.28 km).

During 2013–2016, the model shows negative associations for annual mean temperature (β = -0.97, 95% CI: -1.66 to -0.28), rainfall in the driest quarter (β = -0.66, 95% CI: -1.15 to -0.17), and elevation (β = -0.93, 95% CI: -1.59 to -0.26). Positive associations were observed with livestock (goat) density (β = 0.39, 95% CI: 0.24 to 0.55) and slope (β = 0.31, 95% CI: 0.20 to 0.42). The spatial correlation range was 97.12 km (95% CI: 97.11 to 97.12 km).

For 2017–2020, the model identified negative associations for annual mean temperature (β = -1.31, 95% CI: -1.82 to -0.81), rainfall in the driest quarter (β = -0.91, 95% CI: -1.29 to -0.53), elevation (β = -1.54, 95% CI: -2.03 to -1.05), and livestock (donkey) density (β = -0.19, 95% CI: -0.31 to -0.08). A positive association was seen for slope (β = 0.11, 95% CI: 0.03 to 0.18). The spatial correlation range of 168.64 km (95% CI: 168.64 to 168.64 km).

In the 2021–2024 period, annual mean temperature (β = -1.54, 95% CI: -2.15 to -0.93), rainfall in the driest quarter (β = -1.77, 95% CI: -2.51 to -1.03), and elevation (β = -1.78, 95% CI: -2.42 to -1.14) were negatively associated with prevalence. In contrast, annual rainfall (β = 1.01, 95% CI: 0.41 to 1.60) and livestock (cattle) density (β = 0.74, 95% CI: 0.58 to 0.91) showed positive associations. The spatial correlation range was 180.20 km (95% CI: 180.20 to 180.20 km).

Across these periods, annual mean temperature, rainfall in the driest quarter, and elevation were consistently effective predictors in the models and showed negative associations with onchocerciasis prevalence, with residual spatial correlation increasing overtime (Table 2).

### Effects of co-variates on prevalence of onchocerciasis

**Fig 8:**
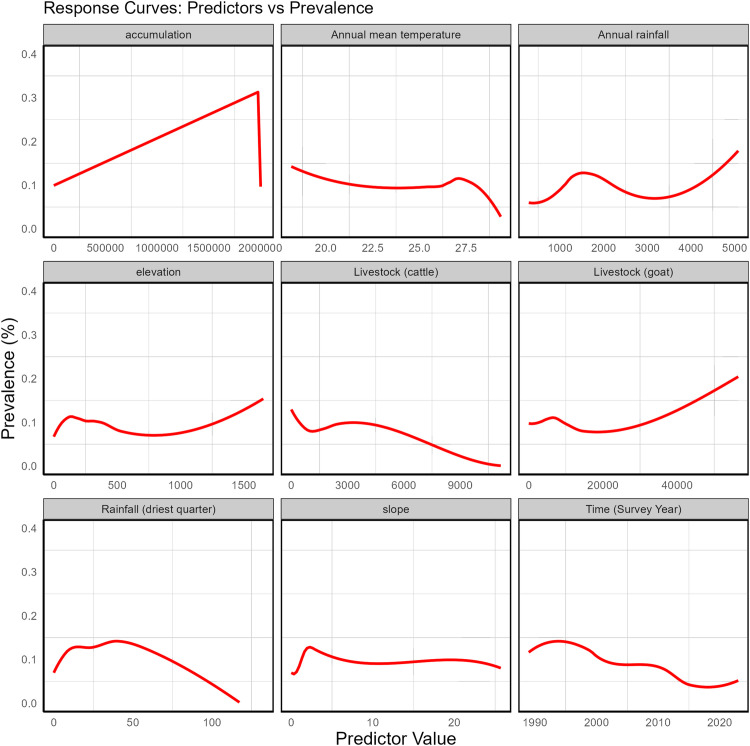
Marginal effects of co-variates on the prevalence of onchocerciasis in Nigeria, using data from all periods (1997–present). The red curves show the posterior mean marginal effects of each covariate from generalized additive model, adjusted for all other variables. Grey shaded bands represent the 95% credible intervals. Effects are presented on the predicted prevalence scale. Increasing or decreasing trends indicate positive or negative associations, respectively, while wider intervals at distributional extremes reflect increased uncertainty due to sparse observations.

Generally, the prevalence of onchocerciasis increased with water accumulation but dropped sharply beyond a certain threshold (Fig 8). Prevalence also increased with elevation, annual rainfall, and goat density, while it decreased with annual mean temperature and cattle density. Minimal rainfall during the driest quarter of the year (approximately up to 50 mm) appeared to be associated with higher prevalence, whereas prevalence declined over time (as the years progressed) (Fig 8).

## Discussion

Nigeria is currently in the elimination phase of onchocerciasis, with 10 endemic States having passed epidemiological assessments and declared eligible to stop mass drug administration (MDA) by the National Onchocerciasis Elimination Committee (NOEC) of the Federal Ministry of Health (FMoH). In this study, we modelled the spatio-temporal prevalence of onchocerciasis across Nigeria to estimate disease risk in unsampled locations, where data are lacking across various years. Missing such cases could, over time, cause setbacks in the progress achieved in the fight against the disease. In many instances, undetected cases could serve as reservoirs of infection, potentially reintroducing transmission to other areas and causing recrudescence in areas previously declared free of onchocerciasis [28]. Previous geostatistical analyses of REMO data primarily focused on delineating pre-control endemicity and identifying populations at risk prior to large-scale ivermectin implementation [29], whereas the present study builds upon this foundation by incorporating multi-period data from the MDA era to examine evolving spatial patterns of prevalence and areas of persistent transmission.

As expected after more than two decades of mass drug administration and other onchocerciasis control efforts across the country, our modelled estimates indicate significant reduction in onchocerciasis prevalence in Nigeria from 1989 to the present. Notably, most States in the northern part of the country were predicted to have experienced a decline from a prevalence of approximately 30% to between 2–5% by 2016. These reductions may be attributable to the long-term and sustained implementation of MDA across the region. Evidence suggests that 12–15 years of consistent ivermectin distribution can achieve elimination in some settings, and many Nigerian States have now implemented MDA for two decades or more. This highlights the critical importance of sustained treatment coverage in reducing transmission and advancing towards elimination [8,30].

However, interpretation of the persistent prevalence observed in parts of southern Nigeria is constrained by the absence of spatially and temporally resolved MDA coverage data. Although some localized studies in southwestern Nigeria have reported suboptimal treatment coverage [4,31], with many endemic communities having received zero rounds of MDA [2,31], these data are insufficient for national inference, limiting our ability to distinguish the relative contributions of coverage gaps and ecological exposure to ongoing transmission. Therefore, future work would benefit from the integration of spatially resolved MDA coverage data from national reporting systems and ESPEN. Improved harmonization of georeferenced coverage data from Nigeria’s NTD programme would enable joint assessment of treatment coverage, ecological exposure, and persistent transmission, strengthening interpretation of residual risk patterns.

Furthermore, more recent prevalence estimates (from 2021 to the present) suggest persistently high levels of onchocerciasis, particularly in cross-border communities along both international (Nigeria–Benin Republic and Nigeria–Cameroon) and interstate boundaries (e.g., Ogun–Oyo, Ondo–Edo, Enugu–Ebonyi, Abia–Cross River). If left unattended, the continued high prevalence in these areas could reverse the progress made in onchocerciasis elimination in Nigeria. Previous study [32] reported onchocerciasis transmission in river systems along Nigeria-Benin Republic border in Southwestern Nigeria. Ayisi et al. [33] highlighted the possible role of sustained Cameroon-Chad cross-border transmission, which is exacerbated by limited cross border coordination in the reintroduction of onchocerciasis in Cameroon, even after 20 years of MDA. A similar situation was reported along the Burkina Faso–Ghana border, where cross-border migration between the two countries has sustained transmission in Burkina Faso, despite significant gains made through both vector control and MDA [8].

Indubitably, effective control and elimination of onchocerciasis in Nigeria will largely depend on coordinated efforts between public health and political sectors, as well as the adequate management of local factors, influences, and challenges that are often overlooked during programme implementation [34]. One of such challenges is the persistent endemicity of onchocerciasis in cross-border regions (as highlighted) in this work, where transmitting vectors, of course, do not recognize/respect borders [8,33,34]. Although national programmes are beginning to pay greater attention to this issue and some level of international cooperation is emerging, these developments remain in their infancy in many countries. A more robust and coordinated approach will be required if onchocerciasis is to be eliminated across Africa [33,34].

In addition to international borders, this study also highlights the need for more active surveillance and adequate interventions in cross-border communities within Nigeria, especially in the southern region of the country. The high prevalence estimates reported around inter-state boundaries buttress the findings by [2] in Enugu State, which reported high onchocerciasis prevalence in two communities in a Local Government Area that shares boundary with Ebonyi State. Enugu was previously declared eligible to stop MDA after passing required epidemiological assessments to confirm transmission had ceased [5,35,36]. Consequently, biannual treatment has resumed in the affected parts of Enugu State as a response to the resurgence of infection. Abia State, which our predictions also show to have high prevalence hotspots in the boundary area with Cross River, was also previously declared eligible to stop MDA like Enugu. Therefore, there is an urgent need to intensify efforts against transmission in cross-border areas, which are often prone to neglect due to poor access, insecurity, low population density, difficult terrain, or weak cross-border coordination [37,38]. An important step in these efforts may include redefining onchocerciasis transmission zones in Nigeria. Presently, the State-level administrative unit is considered a transmission zone for programmatic convenience (e.g., Fig 7) On the other hand, transmission zones should ideally be defined by natural ecological and epidemiological units that reflect geographical areas where *Onchocerca volvulus* transmission by the locally breeding vectors occur (e.g., Fig 6) [39,40]. Effective delineation of the transmission zones could improve the inadequate implementation of interventions in villages located near State administrative boundaries, where the national programme efforts commonly face challenges.

The importance of seasonality in the delivery of interventions for onchocerciasis elimination programmes is well known. This analysis evaluates the impact of climatic, hydrographic, and topographical factors on onchocerciasis prevalence in Nigeria. We identified three environmental factors — mean annual temperature, rainfall in the driest quarter of the year, and elevation — that were consistently associated with onchocerciasis prevalence across time. These variables were consistently negatively associated with prevalence. For example, onchocerciasis prevalence may increase by approximately 2% in areas experiencing a unit decrease in rainfall intensity during the driest quarter of the year (from the tail end of the rainy season to the end of the dry season). Similar patterns were observed for mean annual temperature and elevation. While interpreting the relationship between climatic factors and prevalence of onchocerciasis may sound complex and difficult, our findings can be attributed to the seasonality of blackfly breeding and biting behavior, which are known to be sensitive to changes in temperature, river flow, and rainfall patterns, the key ecological drivers that influence the reproduction, survival and distribution of *Simulium* vectors [33,41–43]. Moreover, dry-season conditions often result in reduced river discharge and create favorable breeding habitats, thereby influencing transmission dynamics [1,44,45].

It is, however, important to note that the observed relationship between climatic factors and the prevalence of onchocerciasis recorded in this study may not be consistent across different regions of the country [46,47], given the persistent effect of climate change and population movement (most often observed at interstate cross-border communities and conflict-affected zones). On the other hand, mean annual rainfall and river flow accumulation were significantly and positively associated with onchocerciasis prevalence in this study, with an approximate 14-unit increase in prevalence observed in locations near rivers with high flow accumulation. This finding is not surprising, as it reinforces the importance of prioritizing first-line communities located near fast-flowing, high-accumulation rivers in efforts to eliminate onchocerciasis [33]. The transmitting vector prefers shaded, rocky, high-energy water bodies — characteristics commonly associated with rivers that exhibit high flow accumulation.

The geostatistical analysis revealed a significant increase in the spatial scale of onchocerciasis prevalence over time, expanding from approximately 32 km (1997–2000) to about 180 km in more recent years (i.e., for any prediction location, its estimated value is influenced by locations up to 180 km away). Although this range is still within the long-distance flight capacity of the vectors (*Simulium* spp.) [48], the widening spatial scale appears plausible, as a study from Nigeria reported a prevalence of up to 26.4% in a local government area previously considered non-endemic for onchocerciasis [4,49]. This trend may be attributed to a combination of ecological and anthropogenic factors, particularly the effects of climate change and human migration [50]. Climate change can expand suitable habitats for *Simulium* blackflies by altering temperature and rainfall patterns, leading to the emergence of new breeding sites in areas that were previously non-endemic [51,52]. Simultaneously, increasing human mobility—driven by conflict, economic hardship, and climate-induced displacement—facilitates the movement of infected individuals into new regions, potentially introducing or reintroducing transmission in areas that were either non-endemic or had previously achieved control, respectively [53–55]. These dynamics may contribute to a broader and more diffuse pattern of transmission, posing challenges to elimination efforts that traditionally target localized endemic foci. Consequently, the observed increase in spatial scale signals the potential need for adaptive, climate-informed surveillance strategies and enhanced regional coordination, particularly in cross-border and conflict-affected areas.

A key limitation of this study concerns the available dataset, particularly the number and distribution of data points for certain time periods (especially 2021 to date). Predictions generated for regions with limited empirical information, such as northern and eastern Nigeria, may therefore be less reliable, as the true prevalence status in these areas might not be fully captured. Addressing this gap will require the collection of additional data to strengthen the accuracy of current and future estimates of onchocerciasis prevalence across the country. Furthermore, this study relies on parasitological indicators derived primarily from skin snip microscopy and, in earlier surveys, nodule palpation. In settings with low transmission intensity or prolonged ivermectin MDA, microfilarial loads may fall below the detection threshold of skin snip microscopy, resulting in potential underestimation of residual infection. Similarly, nodule palpation becomes less specific as endemicity declines and does not reliably capture transmission dynamics in post-control or non-equilibrium settings. Consequently, predicted prevalence in areas nearing elimination may not fully reflect sub-patent or recrudescent transmission. Addressing this limitation will require nationwide surveillance incorporating more sensitive diagnostic tools, such as Ov16 serological assays.

Another limitation is the variation in sampling strategy across time periods. In more recent years, surveys were more concentrated in endemic areas, which may reflect programmatic targeting rather than uniform surveillance. Although the modelling framework accounts for spatial dependence and random effects, preferential sampling could influence predicted prevalence patterns. Future analyses incorporating formal preferential sampling adjustments or sensitivity analyses restricted to consistently sampled areas would further strengthen inference. Nevertheless, despite these data-related constraints, this study provides important insights into the spatial and temporal dynamics of onchocerciasis in Nigeria, offering an evidence base that can guide more targeted interventions and inform priorities for future data collection. Importantly, these limitations also highlight a valuable avenue for future research, emphasizing the need for enhanced surveillance and systematic data collection to refine predictive models and improve control strategies.

## Conclusion

This study demonstrates, for the first time in Nigeria, the use of geostatistical modelling to generate spatio-temporal maps of onchocerciasis prevalence, providing a valuable tool for programmatic decision-making. While significant reductions in disease burden have been achieved, high-prevalence pockets, particularly in cross-border and underserved regions, remain a threat to elimination efforts. Environmental factors, including proximity to fast-flowing rivers and seasonal climatic variations, were found to strongly influence prevalence. These findings underscore the critical value of predictive modelling to strengthen the field surveys in planning and surveillance of the disease. To sustain the progress toward onchocerciasis elimination in Nigeria, there is a need for adaptive, climate-informed strategies, intensified surveillance in high-risk areas, and enhanced coordination—particularly in cross-border and hard-to-reach communities.

While this study focuses on estimating current and historical prevalence patterns, future integration with transmission dynamic models would enable scenario-based projections to further inform elimination planning under varying CDTi strategies. This modelling approach should not be viewed as a one-time exercise but rather as a framework that can be periodically updated, ideally every 3–5 years, in alignment with surveillance and programmatic review cycles. The methodology is transferable to other endemic countries, including smaller settings, provided adequately distributed and quality-assured georeferenced prevalence data are available. Strengthening routine data collection systems will therefore be essential to enable similar spatial analyses to inform elimination planning across diverse epidemiological contexts.

## Supporting information

S1 Appendix Correlation plots of environmental and socio-ecological covariates by survey period. Pearson correlation matrices showing pairwise correlations among climatic, hydrographic, topographic, demographic, and livestock covariates for each survey period: (A) 1997–2000, (B) 2009–2012, (C) 2013–2016, (D) 2017–2020, and (E) 2021–present. Color gradients indicate direction and magnitude of correlations.
(PDF)

S2 Appendix Scatter plots showing relationships between predictors and empirical onchocerciasis prevalence. Scatter plots illustrating associations between environmental and socio-ecological predictors and observed prevalence across (A) overall dataset and stratified by survey periods: (B) 1989–1992, (C) 1993–1996, (D) 1997–2000, (E) 2001–2004, (F) 2005–2008, (G) 2009–2012, (H) 2013–2016, and (I) 2021–present. Red curves represent smoothed trend lines. Table A. Full description of the co-variates codes in the scatter plots.
(PDF)

S3 Appendix Empirical variograms and spatio-temporal prediction maps Fig A. Empirical semivariograms of residuals from period-specific generalized linear models (GLMs) showing spatial autocorrelation for 1997–2000, 2009–2012, 2013–2016, 2017–2020, and 2021–present. Fig B. Model-predicted onchocerciasis prevalence across Nigeria by survey period with corresponding 5% and 95% confidence interval surfaces.
(PDF)

S4 Appendix Supplementary tables and empirical summaries. **Table A.** Summary of empirical survey characteristics and prevalence statistics by survey period, including number of sites, sample size, spatial dispersion, and state coverage.**Table B.** State-level mean empirical prevalence (%) with 95% confidence intervals and number of survey sites by survey period.**Table C.** State-level pairwise comparisons of empirical prevalence (%) across survey periods, including absolute changes, 95% confidence intervals, p-values, and statistical significance. **Table D.** Distribution of states by model-predicted prevalence (%) categories across survey periods. **Table E.** Distribution of southern Nigerian states by model-predicted prevalence (%) categories across survey periods. **Table F.** Linear mixed-effects model results assessing temporal trends in state-level model-predicted prevalence, including fixed effects for survey period, Holm-adjusted pairwise comparisons, and overall temporal effect with state as a random intercept. **Table G.** Pairwise comparisons of model-predicted prevalence (%) across survey periods. **Table H** Overall effect of survey period model-predicted prevalence from linear mixed-effects model.
(PDF)
