# Supplementary material for: Spatio-temporal analysis and geostatistical modelling of onchocerciasis prevalence in Nigeria to support elimination efforts
Source: PLoS Negl Trop Dis. 2026 Mar 9;20(3):e0014090. doi: 10.1371/journal.pntd.0014090 (PMC12981563; doi:10.1371/journal.pntd.0014090)
Supplement: S1 Appendix — Pearson correlation matrices showing pairwise correlations among climatic, hydrographic, topographic, demographic, and livestock covariates for each survey period: (A) 1997–2000, (B) 2009–2012, (C) 2013–2016, (D) 2017–2020, and (E) 2021–present. Color gradients indicate direction and magnitude of correlations. (PDF) [file pntd.0014090.s001.pdf]

Spatio-Temporal Analysis and Geostatistical Modelling of Onchocerciasis Prevalence in Nigeria to Support Elimination Efforts

Ayodele Samuel Babalola[1*], Taiwo A. Adekunle[2], Taiwo P. Babatunde[1], Yasmeen A. Adeniyi[3], Omolola Adeniran[4], Olaitan Omitola[5], Edore Edwin Ito[6], Abiodun Olakiigbe[1], Pam V. Gyang[1], Emeka Makata[4], Babatunde Adewale[1], Olaoluwa P. Akinwale[1], Olufunmilayo A. Idowu[5], Olabanji A. Surakat[2], Adedapo O. Adeogun[1,2], and Monsuru A. Adeleke[2]

S1 Appendix

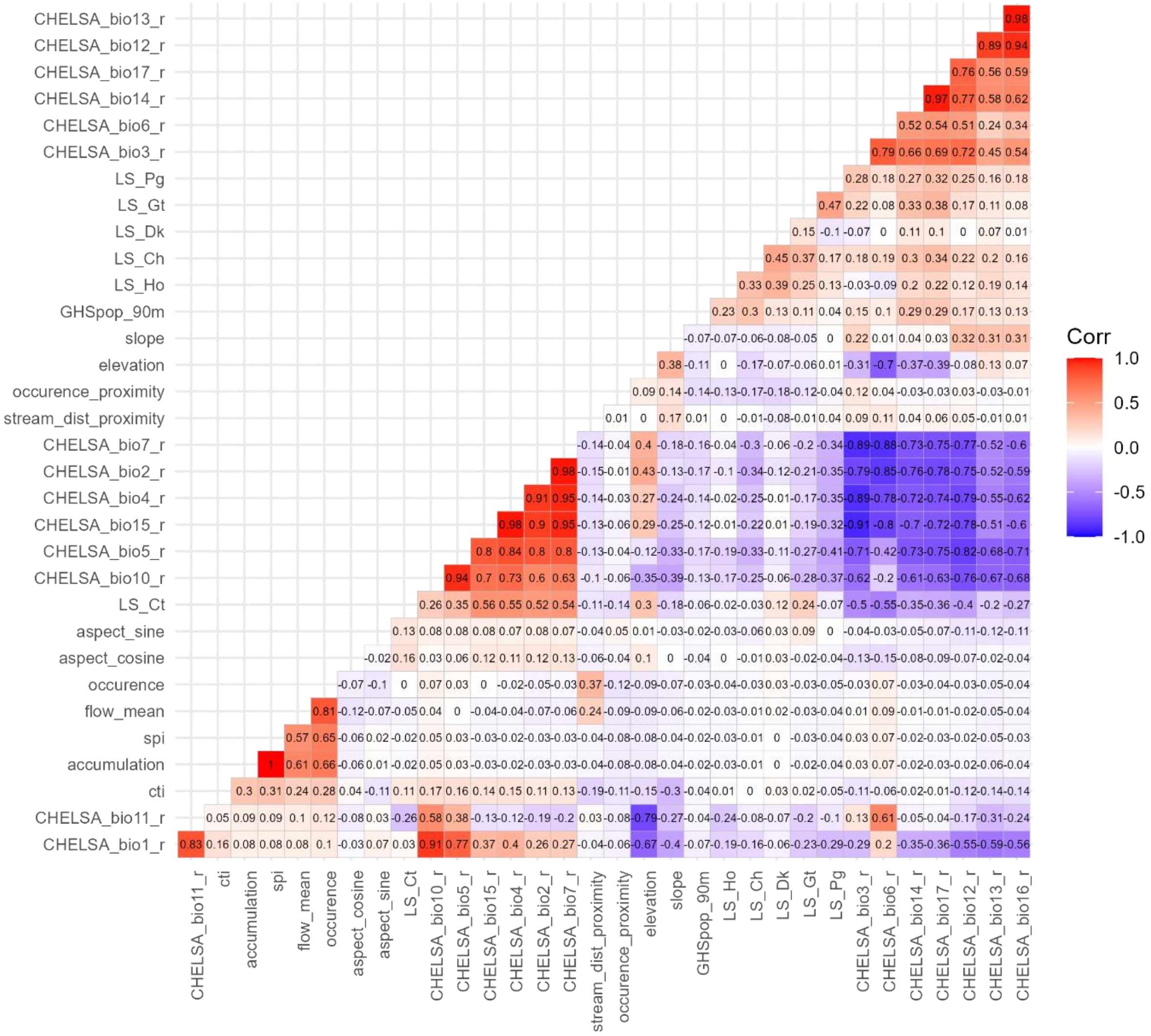

S1 Appendix (Fig. A) correlation plot of the co-variates (1997-2000)

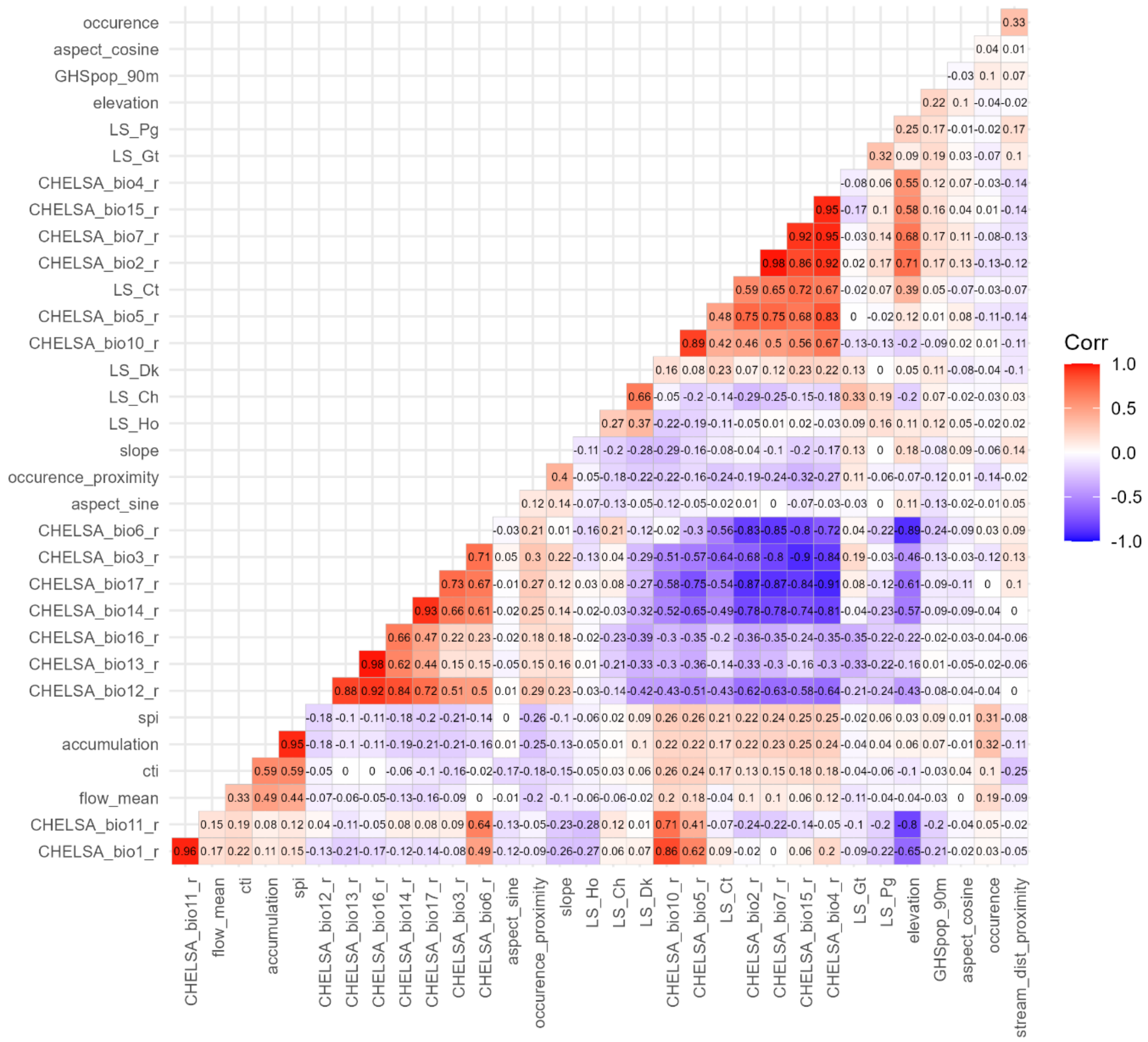

S1 Appendix (Fig. B) correlation plot of the co-variates (2009-2012)

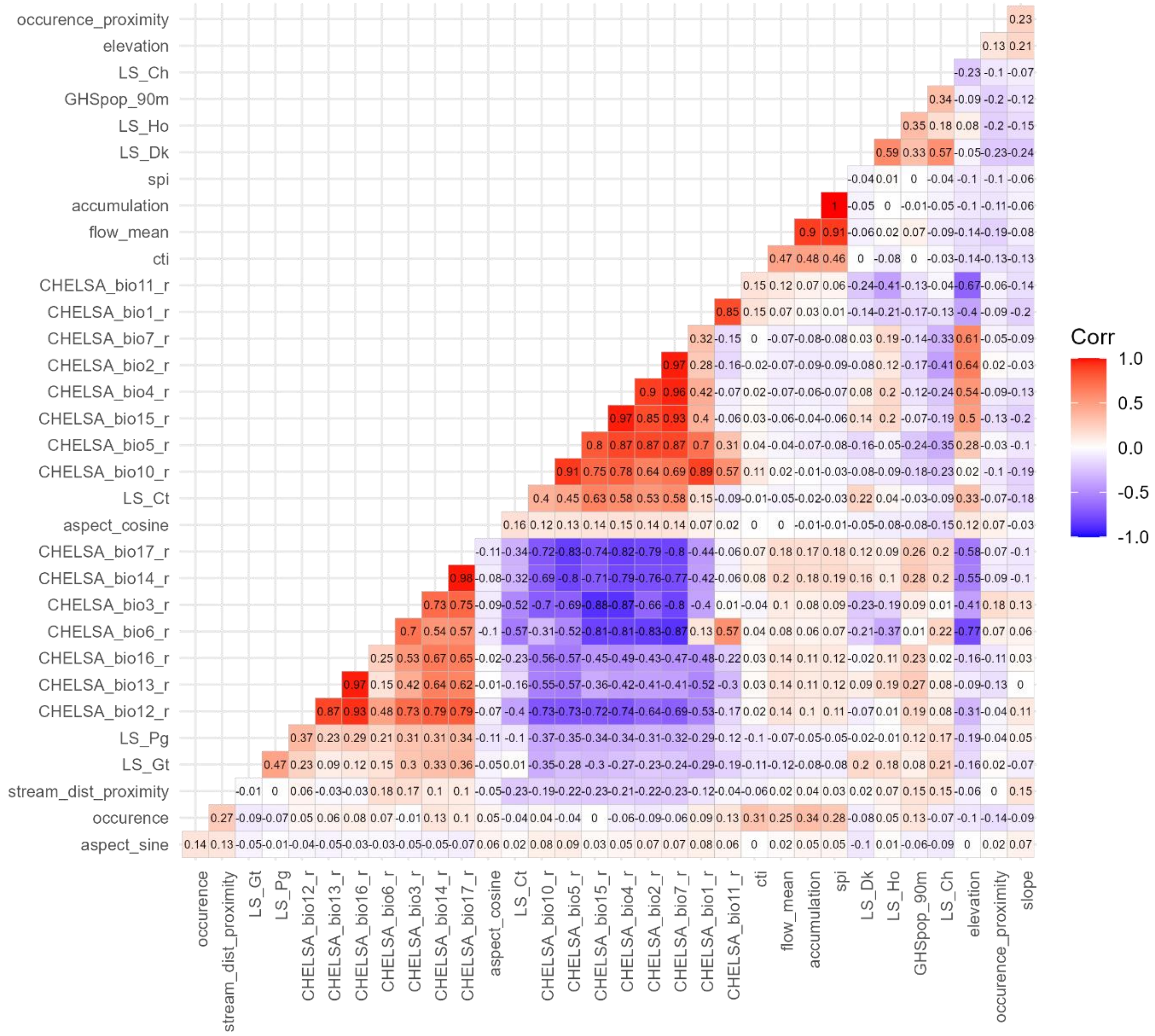

S1 Appendix (Fig. C) correlation plot of the co-variates (2013-2016)

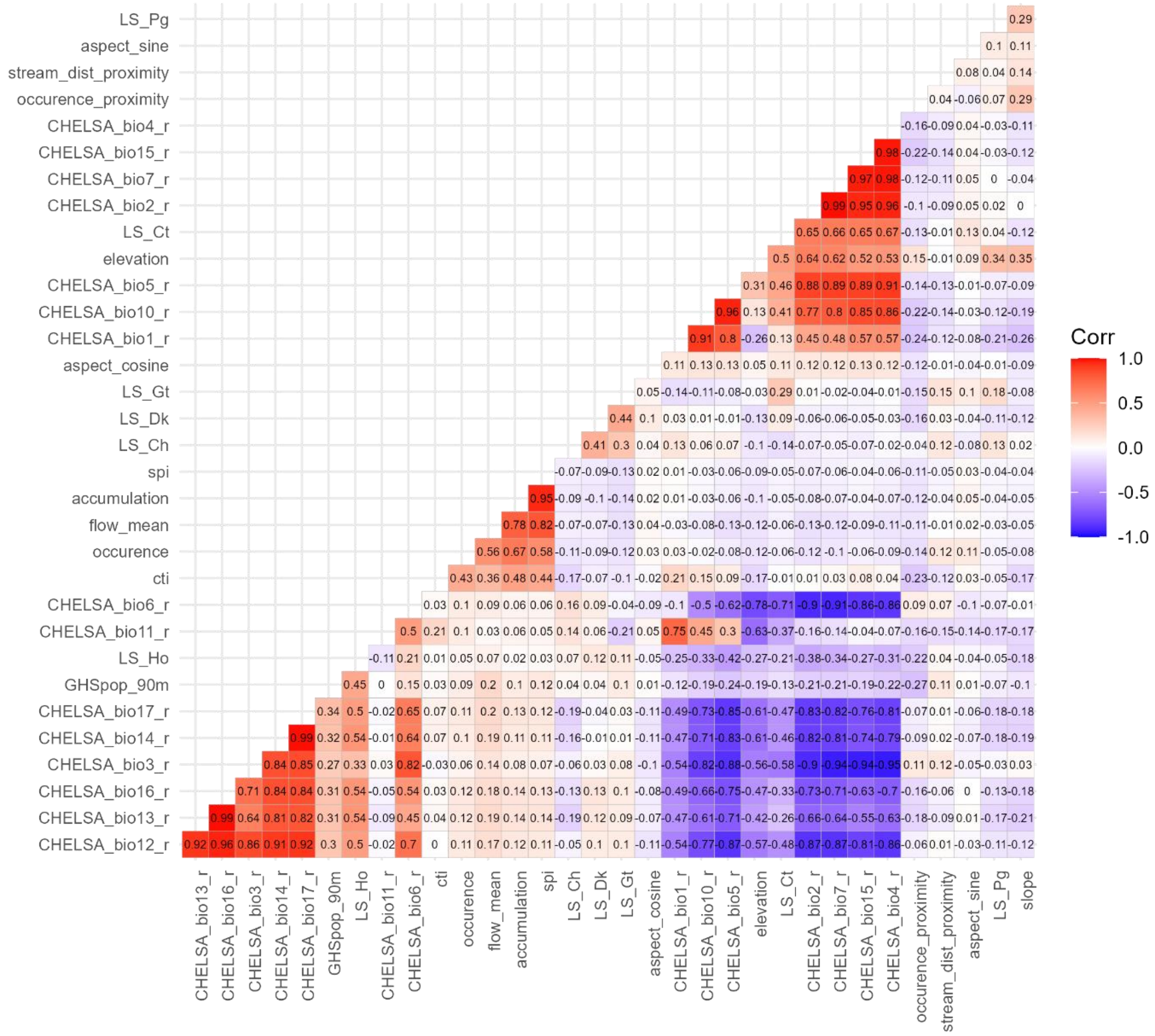

S1 Appendix (Fig. D) correlation plot of the co-variates (2017-2020)

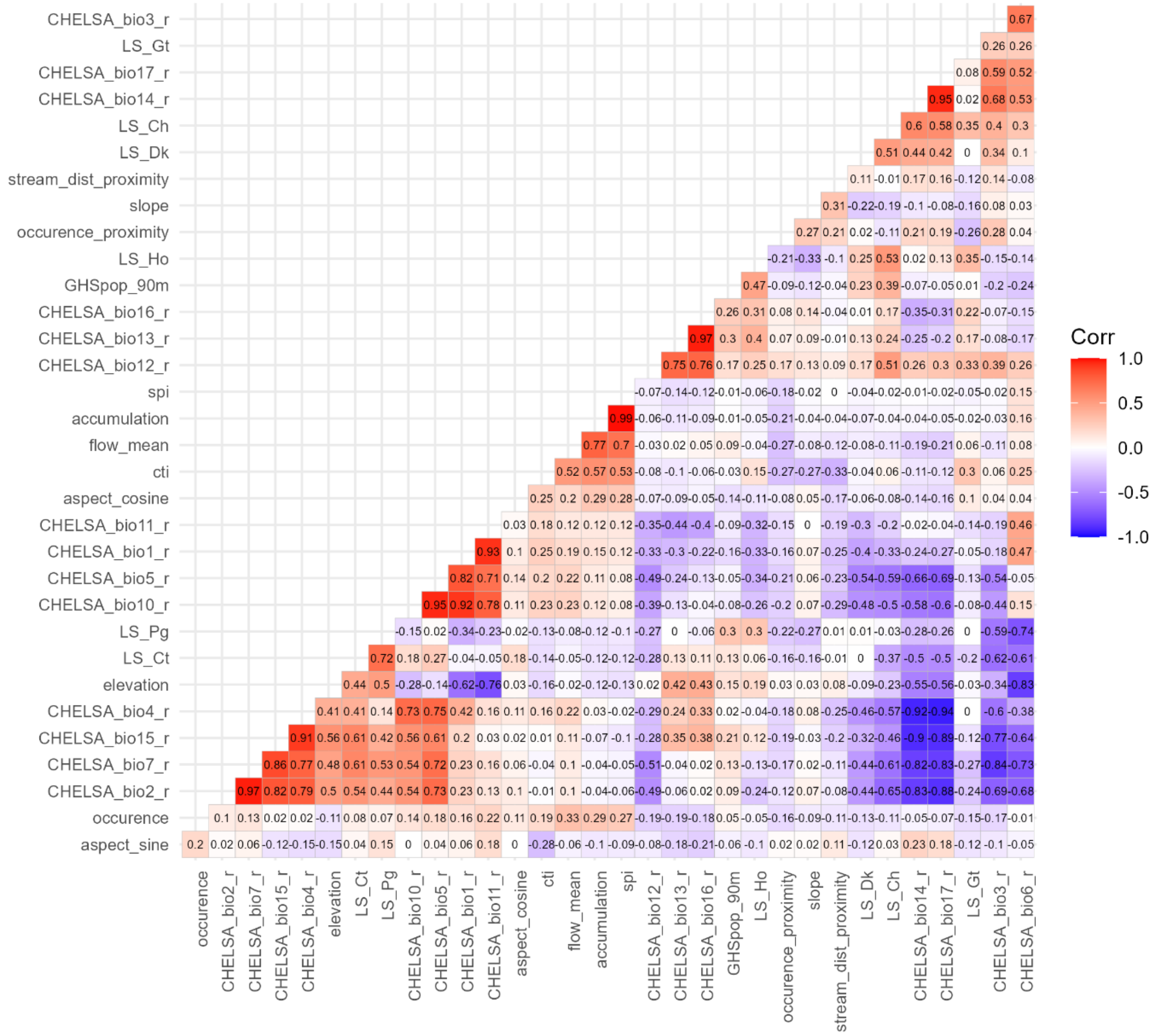

S1 Appendix (Fig. E) correlation plot of the co-variates (2021-date)
