# Supplementary material for: Spatio-temporal analysis and geostatistical modelling of onchocerciasis prevalence in Nigeria to support elimination efforts
Source: PLoS Negl Trop Dis. 2026 Mar 9;20(3):e0014090. doi: 10.1371/journal.pntd.0014090 (PMC12981563; doi:10.1371/journal.pntd.0014090)
Supplement: S2 Appendix — Scatter plots illustrating associations between environmental and socio-ecological predictors and observed prevalence across (A) overall dataset and stratified by survey periods: (B) 1989–1992, (C) 1993–1996, (D) 1997–2000, (E) 2001–2004, (F) 2005–2008, (G) 2009–2012, (H) 2013–2016, and (I) 2021–present. Red curves represent smoothed trend lines. Table A. Full description of the co-variates codes in the scatter plots. (PDF) [file pntd.0014090.s002.pdf]

Spatio-Temporal Analysis and Geostatistical Modelling of Onchocerciasis Prevalence in Nigeria to Support Elimination Efforts

Ayodele Samuel Babalola[1*], Taiwo A. Adekunle[2], Taiwo P. Babatunde[1], Yasmeen A. Adeniyi[3], Omolola Adeniran[4], Olaitan Omitola[5], Edore Edwin Ito[6], Abiodun Olakiigbe[1], Pam V. Gyang[1], Emeka Makata[4], Babatunde Adewale[1], Olaoluwa P. Akinwale[1], Olufunmilayo A. Idowu[5], Olabanji A. Surakat[2], Adedapo O. Adeogun[1,2], and Monsuru A. Adeleke[2]

S2 Appendix

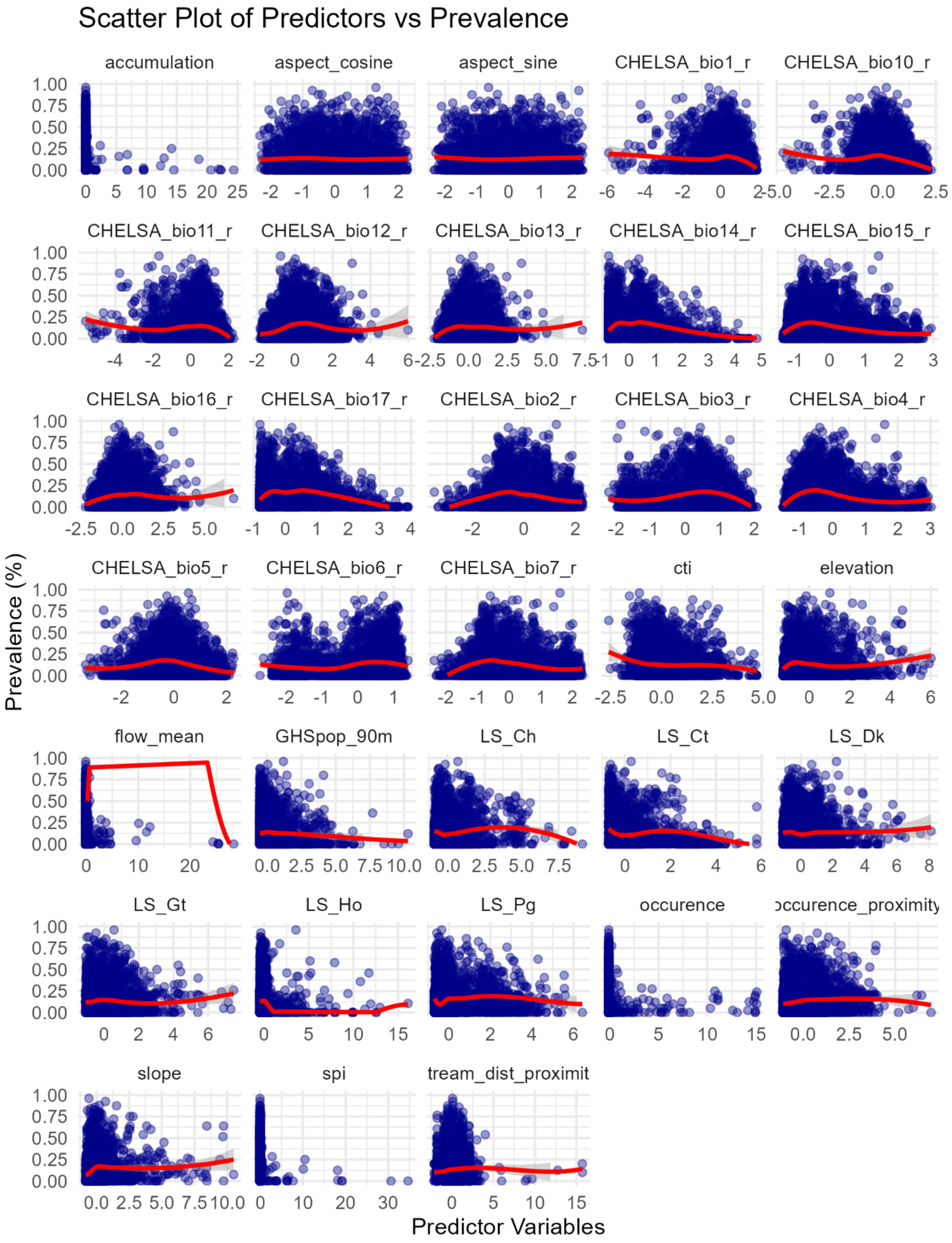

S2 Appendix: (Figure A) Scatter plots showing relationship between predictors and prevalence of oncho in Nigeria (overall data sets)

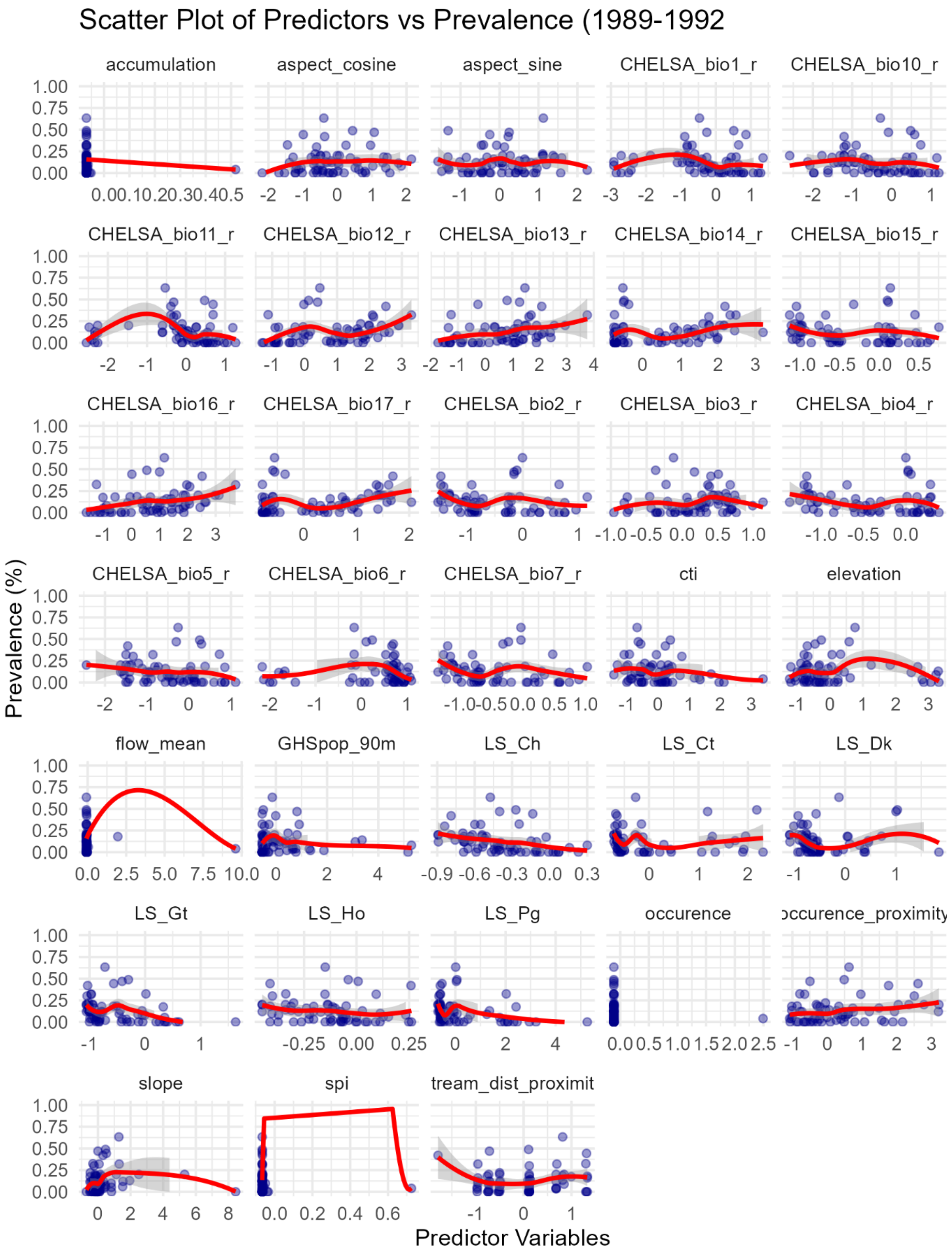

S2 Appendix: (Figure B) Scatter plots showing relationship between predictors and prevalence of oncho in Nigeria (1989-1992 data sets)

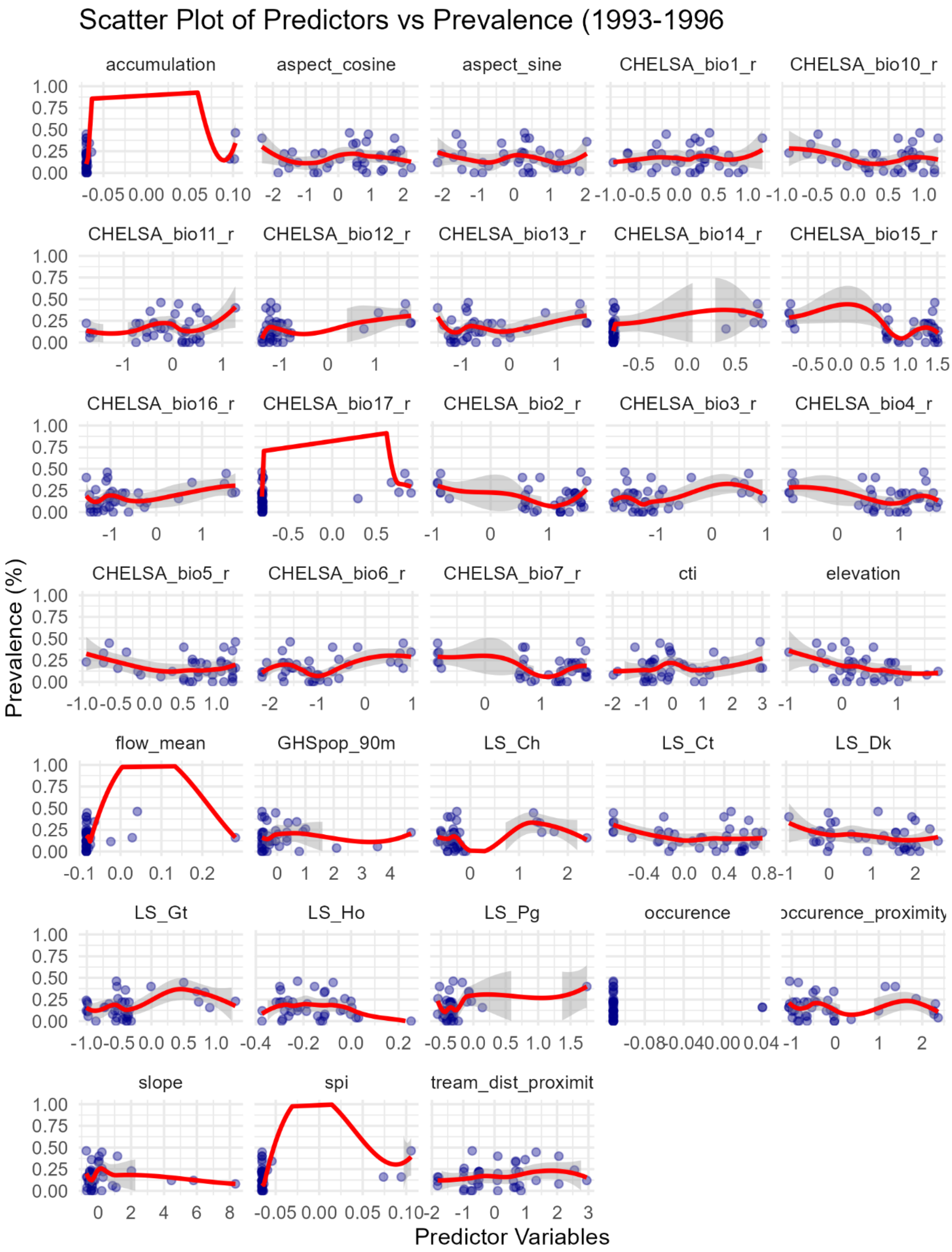

S2 Appendix: (Figure C) Scatter plots showing relationship between predictors and prevalence of oncho in Nigeria (1993-1996 data sets)

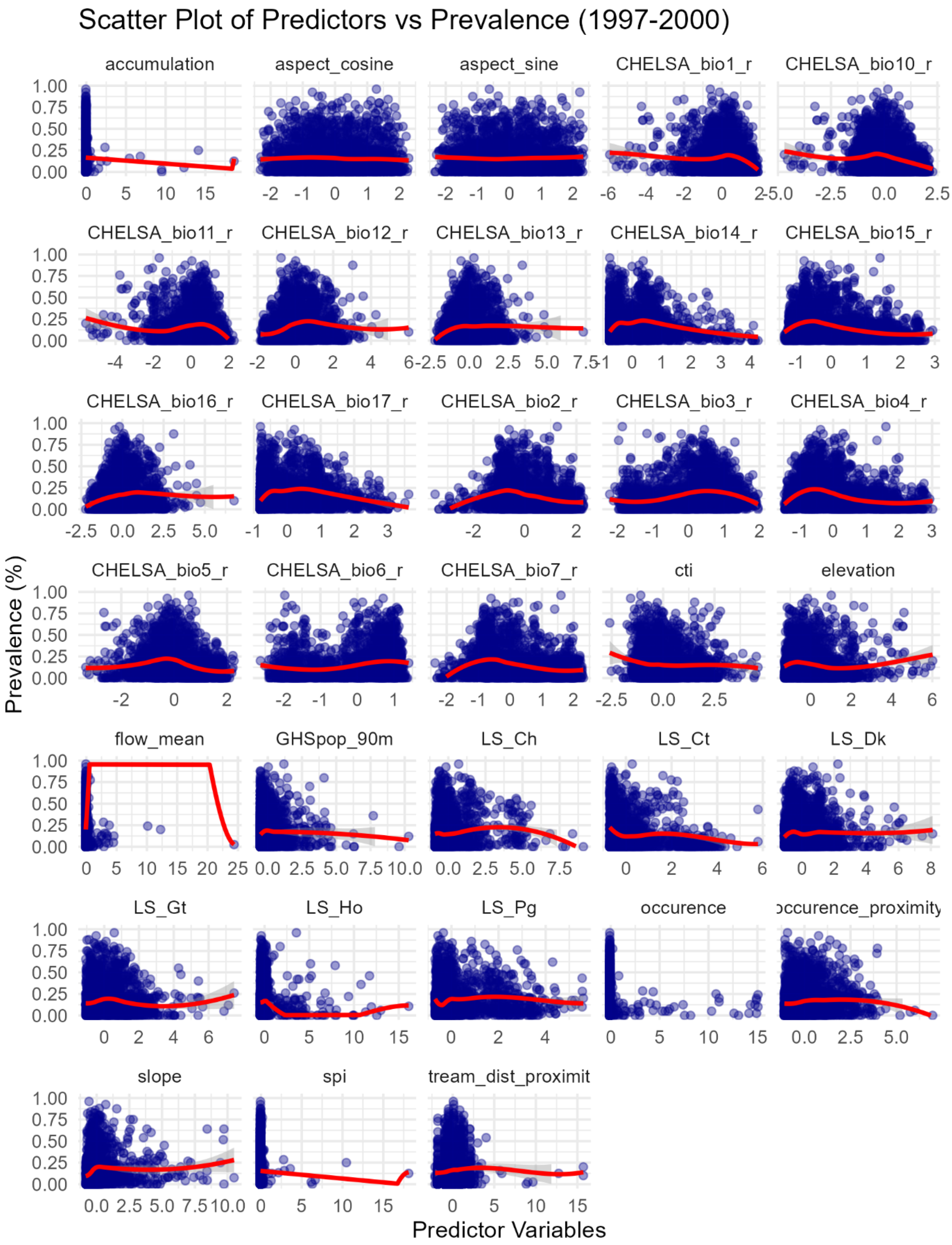

S2 Appendix: (Figure D) Scatter plots showing relationship between predictors and prevalence of oncho in Nigeria (1997-2000 data sets)

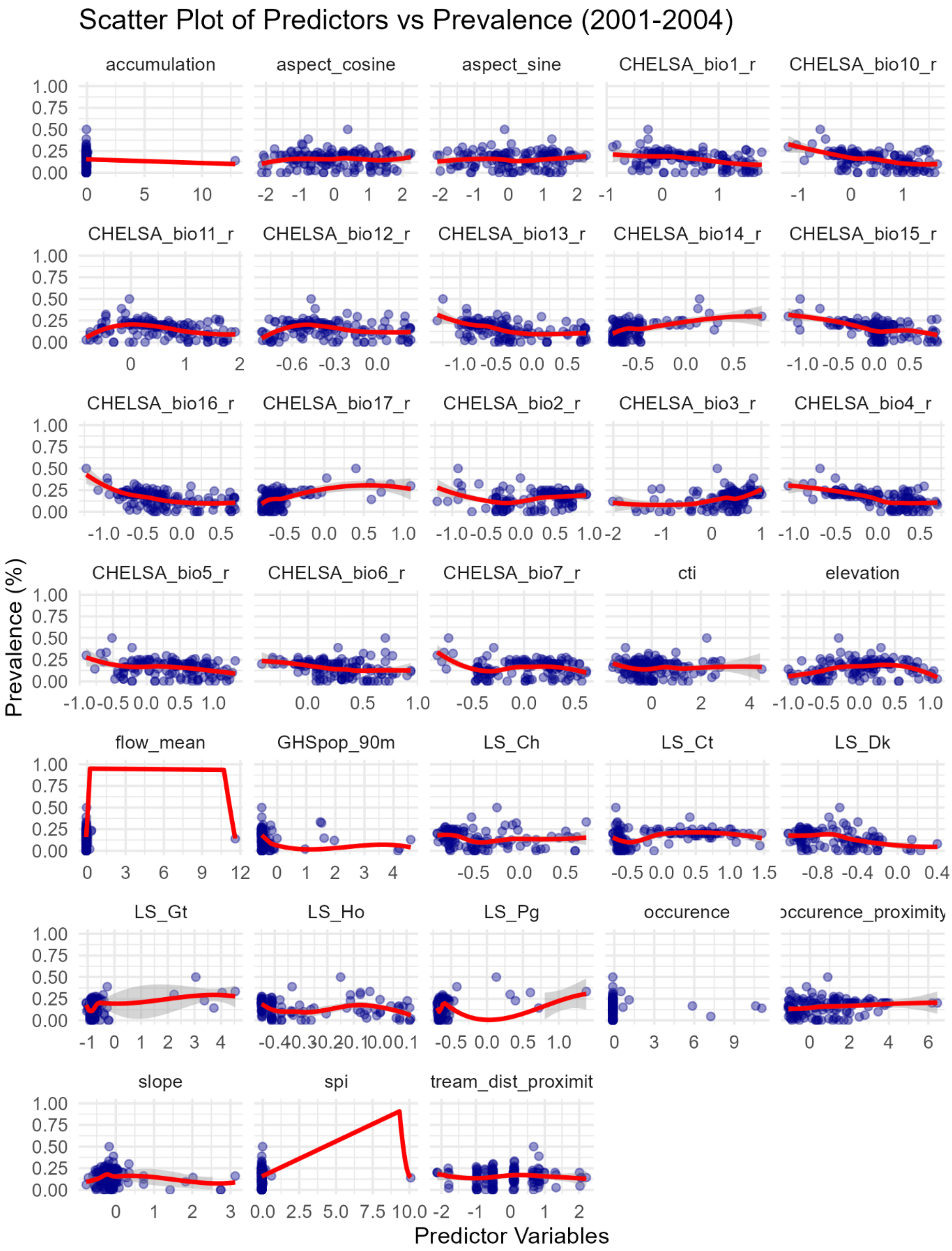

S2 Appendix: (Figure E) Scatter plots showing relationship between predictors and prevalence of oncho in Nigeria (2001-2004 data sets)

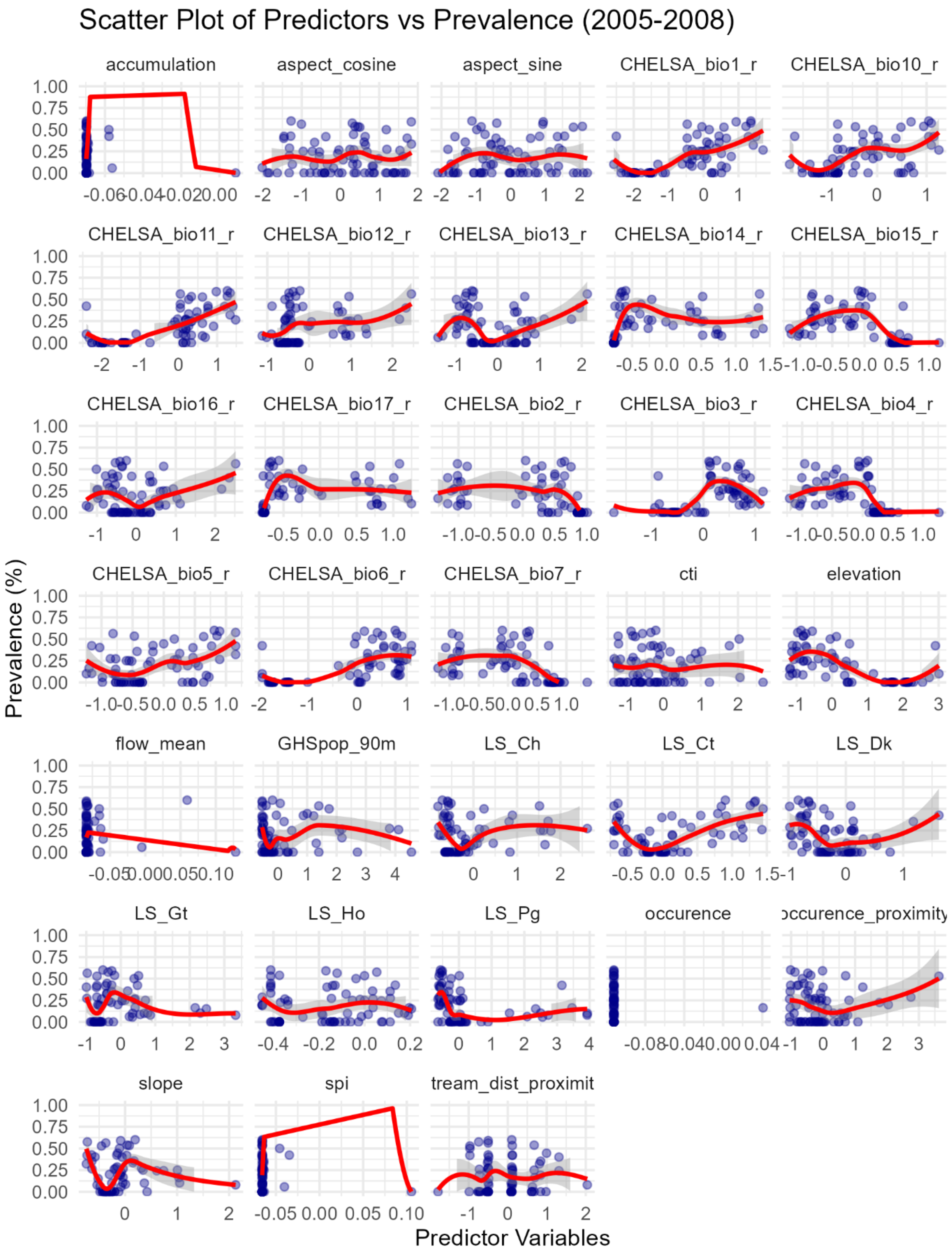

S2 Appendix: (Figure F) Scatter plots showing relationship between predictors and prevalence of oncho in Nigeria (2005-2008 sets)

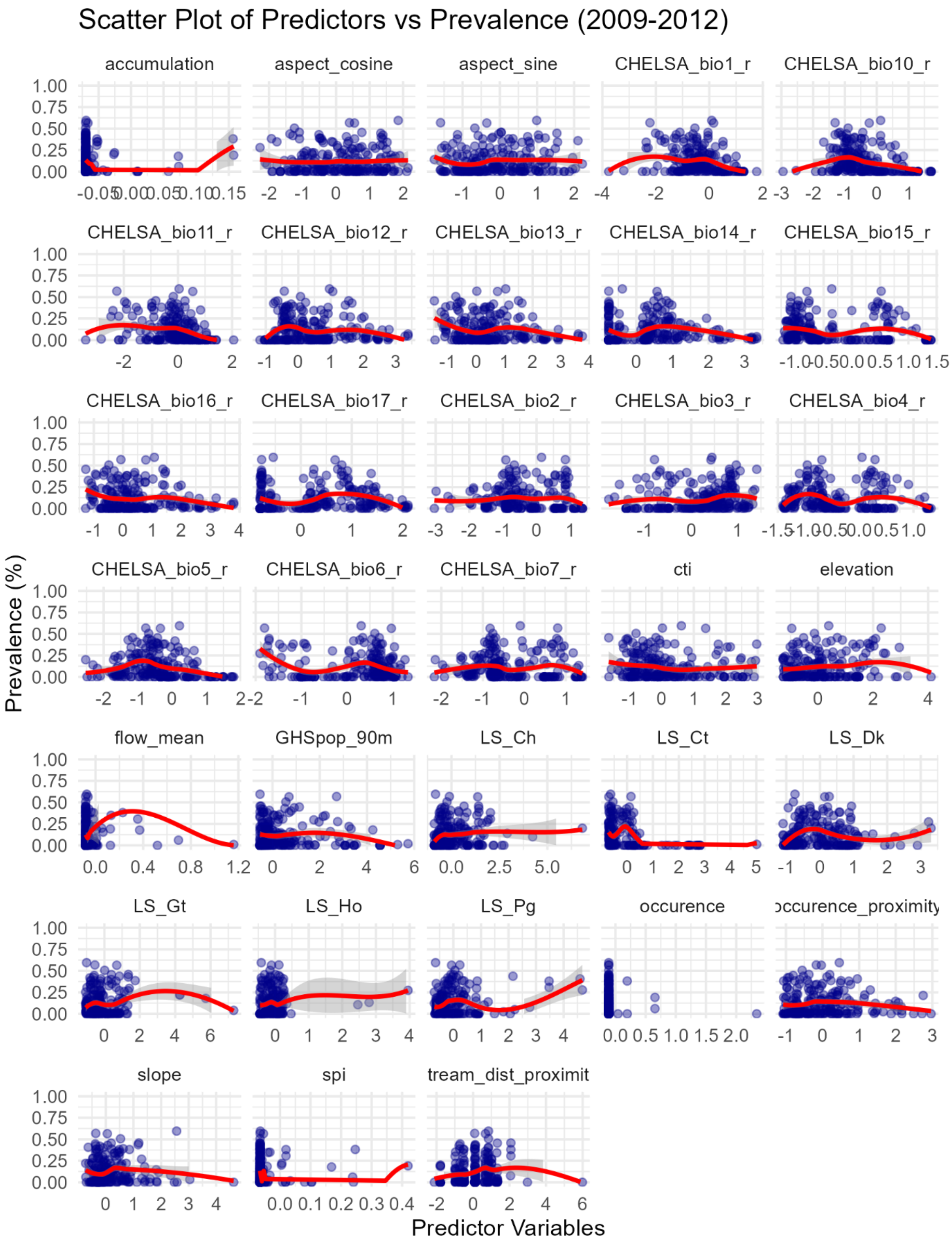

S2 Appendix: (Figure G) Scatter plots showing relationship between predictors and prevalence of oncho in Nigeria (2009-2012 data sets)

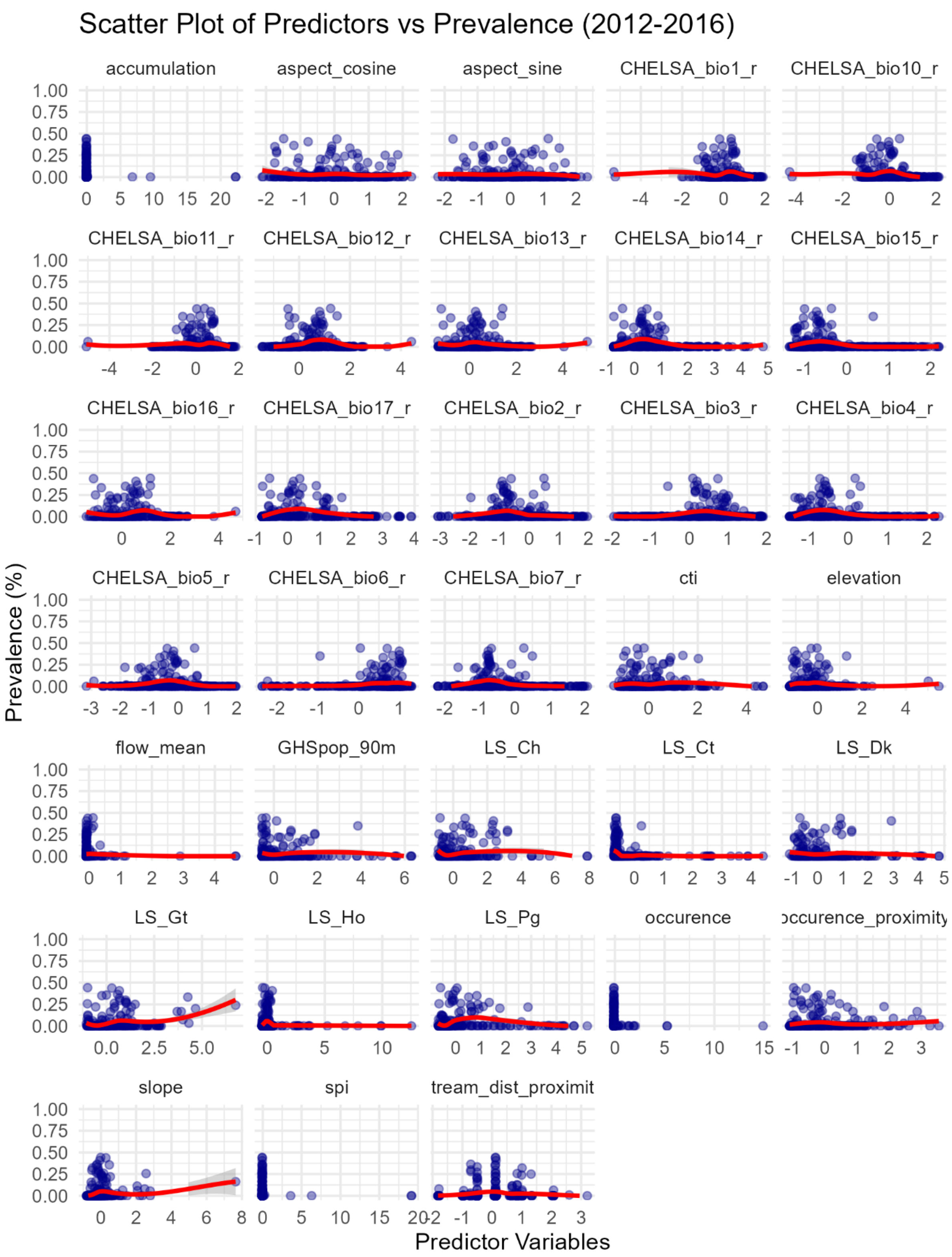

S2 Appendix: (Figure H) Scatter plots showing relationship between predictors and prevalence of oncho in Nigeria (2013-2016 data sets)

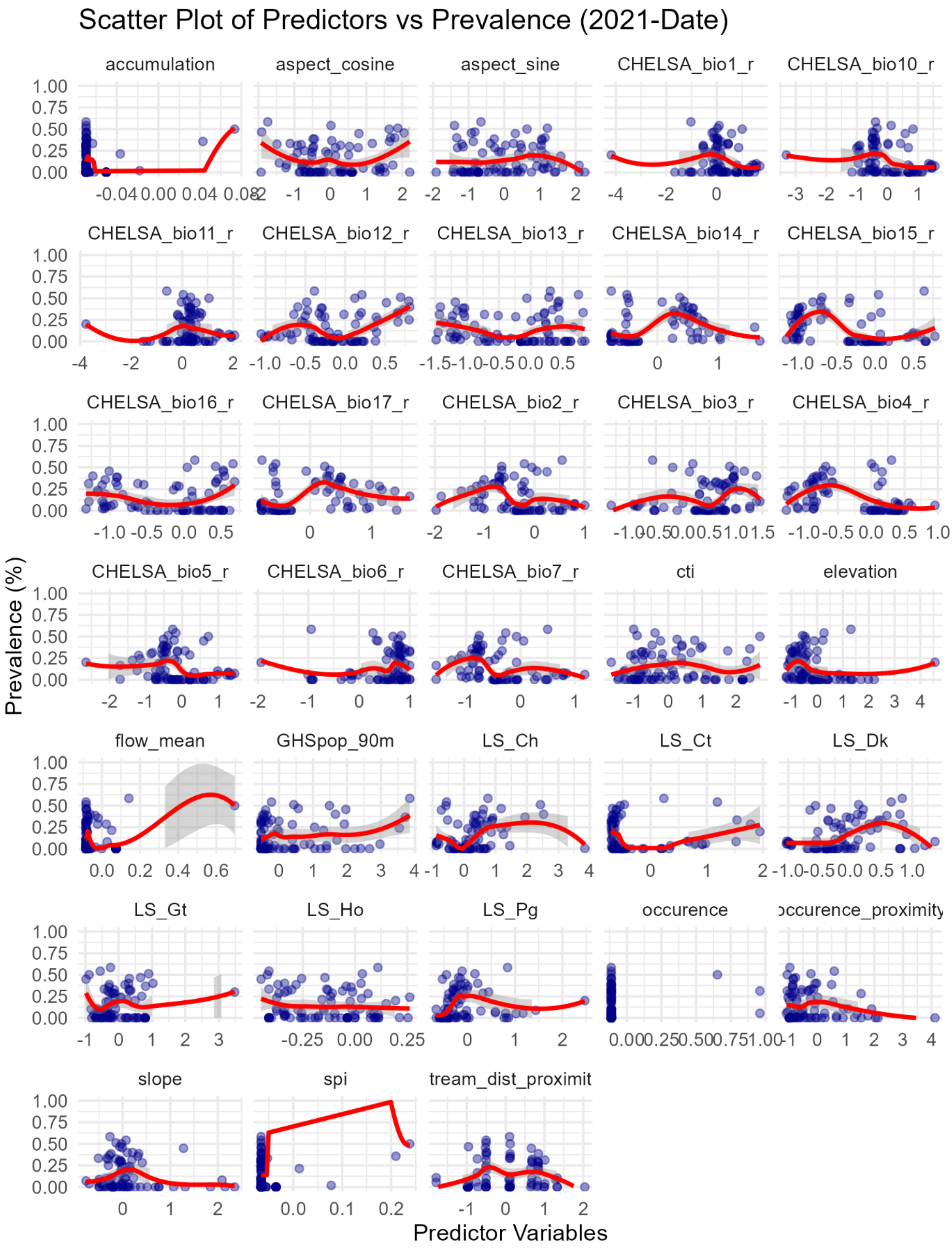

S2 Appendix: (Figure I) Scatter plots showing relationship between predictors and prevalence of oncho in Nigeria (2021-date data sets)

S2 Appendix: Table A. Full description of the co-variates code used in the scatter plots

**Variable Codes**	**Descriptions**
Occurrence	Water occurrence
Accumulation	Water flow accumulation
aspect_cosine	Cosine-transformed terrain aspect (East–West gradient)
aspect_sine	Sine-transformed terrain aspect (North–South gradient)
Chelsa_bio1_r	Annual Mean Temperature (°C)
Chelsa_bio10_r	Mean Temperature of Warmest Quarter (°C)
Chelsa_bio11_r	Mean Temperature of Coldest Quarter (°C)
Chelsa_bio12_r	Annual Precipitation (mm)
Chelsa_bio13_r	Precipitation of Wettest Month (mm)
Chelsa_bio14_r	Precipitation of Driest Month (mm)
Chelsa_bio15_r	Precipitation Seasonality – Coefficient of Variation
Chelsa_bio16_r	Precipitation of Wettest Quarter (mm)
Chelsa_bio17_r	Precipitation of Driest Quarter (mm)
Chelsa_bio2_r	Mean Diurnal Range – Mean monthly (max–min) temperature (°C)
Chelsa_bio3_r	Isothermality (BIO2/BIO7 × 100) (%)
Chelsa_bio4_r	Temperature Seasonality (standard deviation × 100)
Chelsa_bio5_r	Max Temperature of Warmest Month (°C)
Chelsa_bio6_r	Min Temperature of Coldest Month (°C)
Chelsa_bio7_r	Temperature Annual Range (BIO5–BIO6) (°C)
Cti	Compound Topographic Index (Terrain Wetness Index)
Elevation	Elevation above sea level (metres)
Flow_mean	Mean annual stream flow / discharge
GHSpop_90m	Human population count (GHS-POP, 90m resolution)
LS_Ch	Livestock (chicken) density
LS_Ct	Livestock (cattle) density
LS_Dk	Livestock (donkey) density
LS_Gt	Livestock (goat) density
LS_Ho	Livestock (horse) density
LS_Pg	Livestock (pig) density
Occurence_proximity	Proximity to nearest water body
Slope	Terrain slope (degrees)
Spi	Stream power index
Stream_distance_proximity	Euclidean distance to nearest stream
