# Supplementary material for: Spatio-temporal analysis and geostatistical modelling of onchocerciasis prevalence in Nigeria to support elimination efforts
Source: PLoS Negl Trop Dis. 2026 Mar 9;20(3):e0014090. doi: 10.1371/journal.pntd.0014090 (PMC12981563; doi:10.1371/journal.pntd.0014090)
Supplement: S3 Appendix — Empirical semivariograms of residuals from period-specific generalized linear models (GLMs) showing spatial autocorrelation for 1997–2000, 2009–2012, 2013–2016, 2017–2020, and 2021–present. Fig B. Model-predicted onchocerciasis prevalence across Nigeria by survey period with corresponding 5% and 95% confidence interval surfaces. (PDF) [file pntd.0014090.s003.pdf]

Spatio-Temporal Analysis and Geostatistical Modelling of Onchocerciasis Prevalence in Nigeria to Support Elimination Efforts

Ayodele Samuel Babalola[1*], Taiwo A. Adekunle[2], Taiwo P. Babatunde[1], Yasmeen A. Adeniyi[3], Omolola Adeniran[4], Olaitan Omitola[5], Edore Edwin Ito[6], Abiodun Olakiigbe[1], Pam V. Gyang[1], Emeka Makata[4], Babatunde Adewale[1], Olaoluwa P. Akinwale[1], Olufunmilayo A. Idowu[5], Olabanji A. Surakat[2], Adedapo O. Adeogun[1,2], and Monsuru A. Adeleke[2]

S3 Appendix

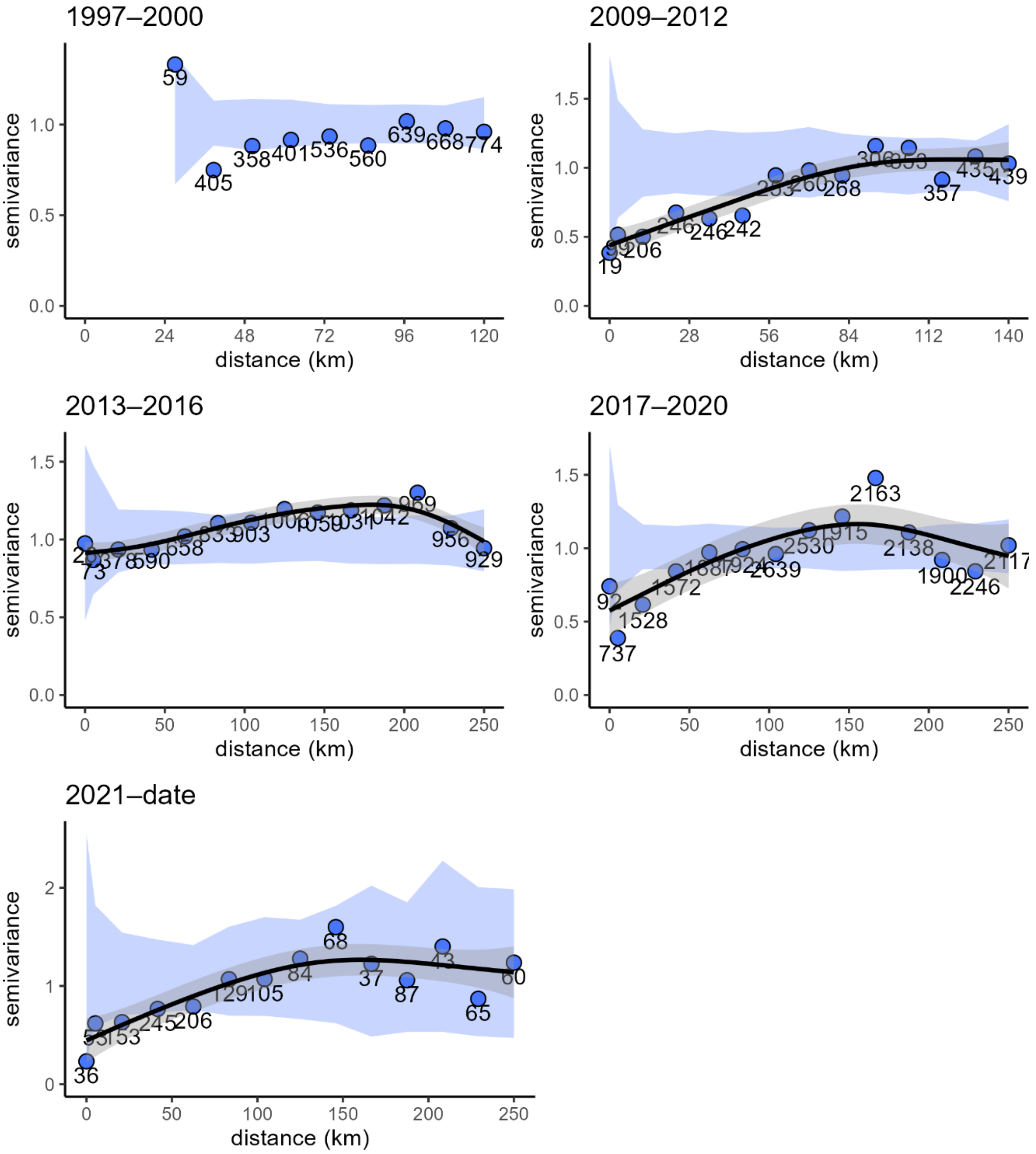

S3 Appendix Figure A: Empirical variograms of GLM fitted for each period

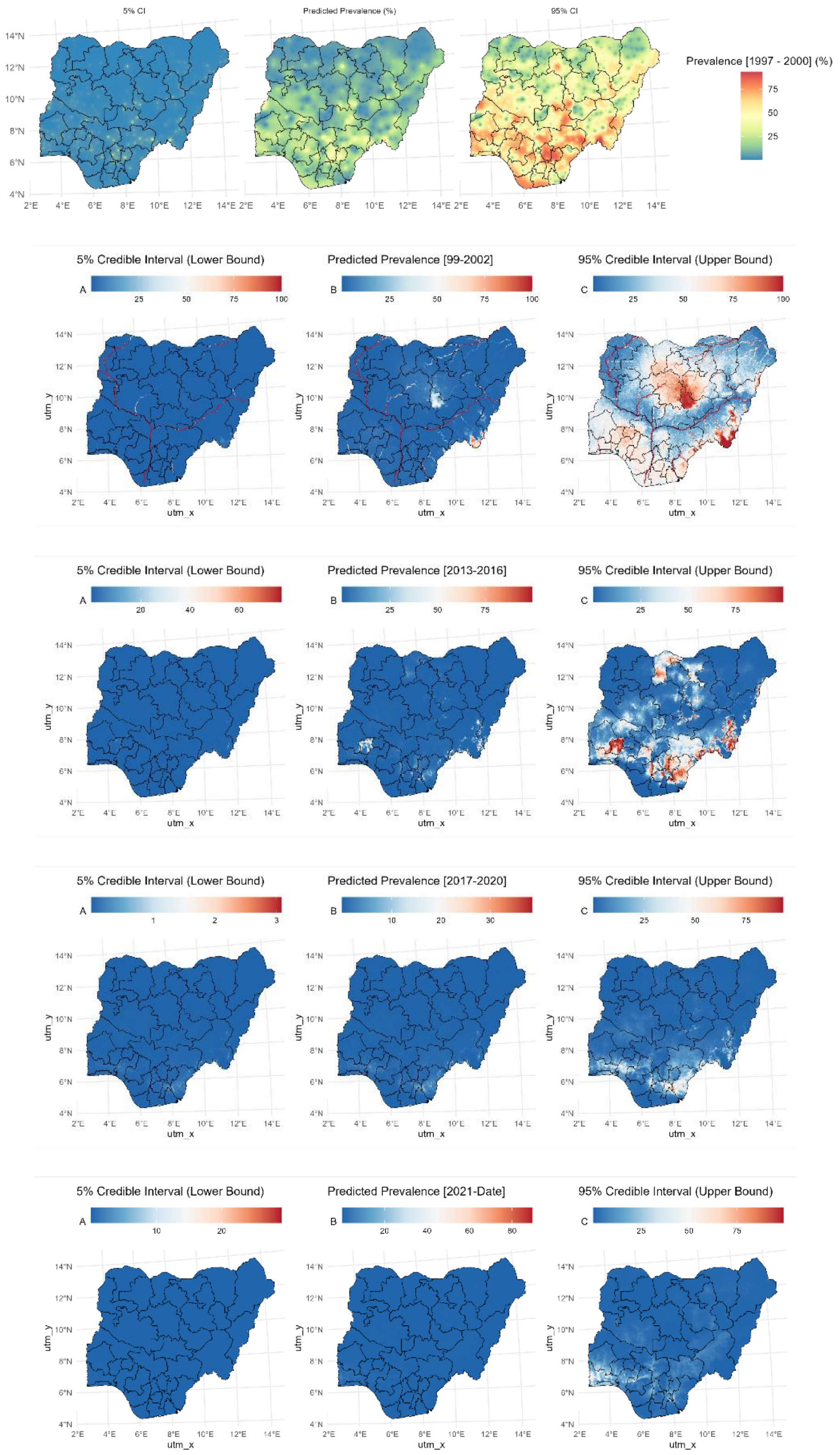

S3 Appendix Figure B: 5% and 95% confidence interval for the spatio-temporal prediction of onchocerciasis in Nigeria

This figure was created by the authors in R programming software (R version 4.1.2, Vienna, Austria). Available at https://www.R-project.org/. The Nigerian shapefile was obtained from

World Bank Data Catalog (https://data.humdata.org/dataset/geoboundaries-admin-boundaries-for-nigeria), an Open license standardized resource of boundaries (i.e., state, county) for every country in the world.
