# Supplementary material for: Spatio-temporal analysis and geostatistical modelling of onchocerciasis prevalence in Nigeria to support elimination efforts
Source: PLoS Negl Trop Dis. 2026 Mar 9;20(3):e0014090. doi: 10.1371/journal.pntd.0014090 (PMC12981563; doi:10.1371/journal.pntd.0014090)
Supplement: S4 Appendix — Table A. Summary of empirical survey characteristics and prevalence statistics by survey period, including number of sites, sample size, spatial dispersion, and state coverage.Table B. State-level mean empirical prevalence (%) with 95% confidence intervals and number of survey sites by survey period.Table C. State-level pairwise comparisons of empirical prevalence (%) across survey periods, including absolute changes, 95% confidence intervals, p-values, and statistical significance. Table D. Distribution of states by model-predicted prevalence (%) categories across survey periods. Table E. Distribution of southern Nigerian states by model-predicted prevalence (%) categories across survey periods. Table F. Linear mixed-effects model results assessing temporal trends in state-level model-predicted prevalence, including fixed effects for survey period, Holm-adjusted pairwise comparisons, and overall temporal effect with state as a random intercept. Table G. Pairwise comparisons of model-predicted prevalence (%) across survey periods. Table H Overall effect of survey period model-predicted prevalence from linear mixed-effects model. (PDF) [file pntd.0014090.s004.pdf]

Spatio-Temporal Analysis and Geostatistical Modelling of Onchocerciasis Prevalence in Nigeria to Support Elimination Efforts

Ayodele Samuel Babalola[1*], Taiwo A. Adekunle[2], Taiwo P. Babatunde[1], Yasmeen A. Adeniyi[3], Omolola Adeniran[4], Olaitan Omitola[5], Edore Edwin Ito[6], Abiodun Olakiigbe[1], Pam V. Gyang[1], Emeka Makata[4], Babatunde Adewale[1], Olaoluwa P. Akinwale[1], Olufunmilayo A. Idowu[5], Olabanji A. Surakat[2], Adedapo O. Adeogun[1,2], and Monsuru A. Adeleke[2]

S4 Appendix

S4 Appendix Table A: Summary of empirical survey characteristics and prevalence statistics by survey period, including number of sites, sample size, spatial dispersion, and state coverage

Period	No. of unique sites	No. of Survey	Total Examined (n)	Mean_ Prevalence	SD	Median_ Prevalence	IQR	No. of States	Median_ interpoint_ (km)	states
1989 - 1992	68	68	4152	12.55	13.73	9.84	16.56	7	19.7	Benue, Cross River, Federal Capital Territory, Kaduna, Nasarawa, Oyo, Plateau
1993 - 1996	41	41	2373	16.85	12.87	15.71	16.22	4	12.3	Bauchi, Enugu, Gombe, Plateau
1997 - 2000	2733	2764	170092	15.72	16.74	10	19.5	37	10.2	Abia, Adamawa, Akwa Ibom, Anambra, Bauchi, Bayelsa, Benue, Borno, Cross River, Delta, Ebonyi, Edo, Ekiti, Enugu, Federal Capital Territory, Gombe, Imo, Jigawa, Kaduna, Kano, Katsina, Kebbi, Kogi, Kwara, Lagos, Nasarawa, Niger, Ogun, Ondo, Osun, Oyo, Plateau, Rivers, Sokoto, Taraba, Yobe, Zamfara
2001 - 2004	135	136	5315	15.37	9.07	17.08	10.98	5	13.5	Federal Capital Territory, Kebbi, Kwara, Niger, Osun
2005 - 2008	75	75	21238	18.13	19.19	10.3	30.42	7	9.5	Akwa Ibom, Anambra, Cross River, Kaduna, Kogi, Kwara, Plateau
2009 - 2012	215	229	40181	11.64	14.05	5.47	18.49	20	16.1	Abia, Adamawa, Cross River, Ebonyi, Edo, Ekiti, Enugu, Kaduna, Kogi, Kwara, Lagos, Nasarawa, Niger, Ogun, Ondo, Osun, Oyo, Plateau, Taraba, Zamfara
2013 - 2016	285	311	46511	3.02	8.1	0	0	28	23.1	Abia, Akwa Ibom, Anambra, Bauchi, Bayelsa, Benue, Cross River, Delta, Ebonyi, Edo, Enugu, Federal Capital Territory, Imo, Jigawa, Kaduna, Kano, Kebbi, Kogi, Kwara, Lagos, Niger, Ogun, Osun, Oyo, Rivers, Sokoto, Taraba, Zamfara
2017 - 2020	396	435	38919	1.67	5.44	0	0	19	13.2	Abia, Bauchi, Bayelsa, Cross River, Delta, Ebonyi, Edo, Jigawa, Kano, Kebbi, Nasarawa, Niger, Ogun, Oyo, Plateau, Rivers, Sokoto, Yobe, Zamfara
2021 - date	81	89	10141	13.92	16.8	5.42	28.57	10	17	Adamawa, Enugu, Federal Capital Territory, Kaduna, Nasarawa, Niger, Ogun, Oyo, Plateau, Taraba

S4 Appendix Table B: Table. State-level mean empirical prevalence (%) with 95% confidence intervals and number of survey sites, by survey period in Nigeria

State	Period	No. of sites	Mean_Prevalence	CI_95
Abia	1997 - 2000	39	24.29	19.03-29.56
Abia	2009 - 2012	4	34.28	28.34-40.23
Abia	2013 - 2016	13	0.37	-0.36-1.1
Abia	2017 - 2020	3	0.18	-0.17-0.54
Adamawa	1997 - 2000	66	4.71	3.4-6.01
Adamawa	2009 - 2012	10	0	0-0
Adamawa	2021 - date	8	6.17	5-7.34
Akwa Ibom	1997 - 2000	77	8.39	6.48-10.29
Akwa Ibom	2005 - 2008	5	11.98	8.81-15.16
Akwa Ibom	2013 - 2016	13	0.1	-0.03-0.23
Anambra	1997 - 2000	36	29.57	21.21-37.93
Anambra	2005 - 2008	9	30.81	22.48-39.13
Anambra	2013 - 2016	2	0	0-0
Bauchi	1993 - 1996	8	13.31	7.55-19.07
Bauchi	1997 - 2000	66	11.85	8.64-15.06
Bauchi	2013 - 2016	5	0	0-0
Bauchi	2017 - 2020	18	0	0-0
Bayelsa	1997 - 2000	2	16.25	13.8-18.7
Bayelsa	2013 - 2016	6	0	0-0
Bayelsa	2017 - 2020	20	0.05	-0.05-0.15
Benue	1989 - 1992	1	0	NA-NA
Benue	1997 - 2000	92	31.47	27.21-35.73
Benue	2013 - 2016	9	5.17	-0.37-10.72
Borno	1997 - 2000	69	12.33	9.97-14.69
Cross River	1989 - 1992	37	12.7	9.43-15.98
Cross River	1997 - 2000	74	10.94	7.88-14
Cross River	2005 - 2008	3	41.22	24.71-57.74
Cross River	2009 - 2012	43	5.3	3.79-6.82
Cross River	2013 - 2016	1	0	NA-NA
Cross River	2017 - 2020	1	0	NA-NA
Delta	1997 - 2000	29	19.14	12.41-25.87
Delta	2013 - 2016	17	2.02	-0.03-4.06
Delta	2017 - 2020	56	3.07	1.4-4.73
Ebonyi	1997 - 2000	45	20.96	14.64-27.27
Ebonyi	2009 - 2012	16	3.38	-0.99-7.75
Ebonyi	2013 - 2016	14	18.26	9.86-26.66
Ebonyi	2017 - 2020	40	0.83	0.18-1.49
Edo	1997 - 2000	55	23.45	19.45-27.46
Edo	2009 - 2012	4	34.48	21.62-47.33
Edo	2013 - 2016	8	0	0-0
Edo	2017 - 2020	52	1.83	0.84-2.81
Ekiti	1997 - 2000	41	15.6	12.31-18.88

Ekiti	2009 - 2012	7	2.61	-0.59-5.8
Enugu	1993 - 1996	6	28.82	20.45-37.18
Enugu	1997 - 2000	58	32.88	26.21-39.55
Enugu	2009 - 2012	6	35.51	23.07-47.96
Enugu	2013 - 2016	4	23.66	14.63-32.68
Enugu	2021 - date	7	37.83	31.2-44.45
FCT	1989 - 1992	1	44.26	NA-NA
FCT	1997 - 2000	44	8.68	4.61-12.75
FCT	2001 - 2004	2	0	0-0
FCT	2013 - 2016	10	0.29	-0.28-0.86
FCT	2021 - date	30	0	0-0
Gombe	1993 - 1996	26	14.28	9.46-19.1
Gombe	1997 - 2000	55	16.08	12.6-19.56
Imo	1997 - 2000	77	20.81	17.23-24.38
Imo	2013 - 2016	3	0	0-0
Jigawa	1997 - 2000	45	4.62	2.79-6.45
Jigawa	2013 - 2016	1	0	NA-NA
Jigawa	2017 - 2020	27	0	0-0
Kaduna	1989 - 1992	1	11.43	NA-NA
Kaduna	1997 - 2000	198	15.13	12.39-17.88
Kaduna	2005 - 2008	29	0	0-0
Kaduna	2009 - 2012	40	19.21	13.97-24.46
Kaduna	2013 - 2016	17	2.06	-1.98-6.09
Kaduna	2021 - date	6	9.72	-9.33-28.78
Kano	1997 - 2000	88	13.57	9.71-17.43
Kano	2013 - 2016	12	0	0-0
Kano	2017 - 2020	3	0	0-0
Katsina	1997 - 2000	124	3.17	2.43-3.91
Kebbi	1997 - 2000	94	6.97	5.51-8.42
Kebbi	2001 - 2004	3	4.18	-0.36-8.72
Kebbi	2013 - 2016	20	0	0-0
Kebbi	2017 - 2020	45	0.24	0.06-0.43
Kogi	1997 - 2000	212	21.87	19.36-24.37
Kogi	2005 - 2008	1	26.5	NA-NA
Kogi	2009 - 2012	12	7.56	3.95-11.17
Kogi	2013 - 2016	4	0.2	-0.19-0.59
Kwara	1997 - 2000	131	19.13	16.47-21.8
Kwara	2001 - 2004	59	20.72	19.63-21.81
Kwara	2005 - 2008	23	34.78	28.5-41.07
Kwara	2009 - 2012	2	11.7	-11.23-34.64
Kwara	2013 - 2016	12	1.32	-0.52-3.17
Lagos	1997 - 2000	15	4	1.35-6.65
Lagos	2009 - 2012	1	0	NA-NA
Lagos	2013 - 2016	8	0.08	-0.08-0.23
Nasarawa	1989 - 1992	11	20.18	6.88-33.47
Nasarawa	1997 - 2000	80	21.99	16.8-27.17
Nasarawa	2009 - 2012	6	0	0-0
Nasarawa	2017 - 2020	15	0.04	-0.04-0.13

Nasarawa	2021 - date	4	31.1	12.66-49.54
Niger	1997 - 2000	158	14.14	12.94-15.35
Niger	2001 - 2004	66	10.19	8.39-12
Niger	2009 - 2012	2	0	0-0
Niger	2013 - 2016	15	0	0-0
Niger	2017 - 2020	1	5.33	NA-NA
Niger	2021 - date	1	10	NA-NA
Ogun	1997 - 2000	52	14.22	11.25-17.18
Ogun	2009 - 2012	23	12.48	9.09-15.88
Ogun	2013 - 2016	20	2.06	-0.19-4.3
Ogun	2017 - 2020	24	16.86	12-21.72
Ogun	2021 - date	21	16.42	11.51-21.32
Ondo	1997 - 2000	71	18.33	15.98-20.68
Ondo	2009 - 2012	4	16.59	-1.27-34.45
Osun	1997 - 2000	60	17.63	13.88-21.38
Osun	2001 - 2004	6	30.41	20.85-39.98
Osun	2009 - 2012	5	17.47	10.71-24.23
Osun	2013 - 2016	11	15.93	10.36-21.51
Oyo	1989 - 1992	1	8	NA-NA
Oyo	1997 - 2000	107	18.33	16.45-20.22
Oyo	2009 - 2012	17	22.56	16.53-28.59
Oyo	2013 - 2016	21	4.2	-0.37-8.77
Oyo	2017 - 2020	37	0.11	-0.04-0.26
Oyo	2021 - date	9	30.22	21.57-38.87
Plateau	1989 - 1992	16	6.13	1.46-10.79
Plateau	1993 - 1996	1	40	NA-NA
Plateau	1997 - 2000	59	19.32	13.37-25.28
Plateau	2005 - 2008	5	14.45	0.73-28.18
Plateau	2009 - 2012	4	0.3	-0.29-0.88
Plateau	2017 - 2020	23	0.02	-0.02-0.07
Plateau	2021 - date	1	20	NA-NA
Rivers	1997 - 2000	10	9.6	5.08-14.12
Rivers	2013 - 2016	10	0	0-0
Rivers	2017 - 2020	22	0	0-0
Sokoto	1997 - 2000	37	1.62	0.65-2.59
Sokoto	2013 - 2016	1	0	NA-NA
Sokoto	2017 - 2020	34	0	0-0
Taraba	1997 - 2000	167	15.64	12.97-18.31
Taraba	2009 - 2012	12	14	4.41-23.58
Taraba	2013 - 2016	43	3.27	0.88-5.66
Taraba	2021 - date	2	47.41	42.34-52.48
Yobe	1997 - 2000	81	12.53	10.68-14.37
Yobe	2017 - 2020	2	0	0-0
Zamfara	1997 - 2000	50	5.55	3.7-7.39
Zamfara	2009 - 2012	11	0	0-0
Zamfara	2013 - 2016	11	0	0-0
Zamfara	2017 - 2020	12	0	0-0

S4 Appendix Table C: State-level pairwise comparisons of mean empirical prevalence (%) across survey periods, including 95% confidence intervals, absolute change, and p-values.

State	Period	Previous_Period	Mean_Prevalence	CI_95	Change_in_Prevalence	p_value	Significance
Abia	2009 - 2012	1997 - 2000	34.3	28.34 - 40.23	9.99	0.036	*
Abia	2013 - 2016	2009 - 2012	0.4	-1.46	-33.91	0.001	**
Abia	2017 - 2020	2013 - 2016	0.2	-0.71	-0.19	0.652	ns
Adamawa	2009 - 2012	1997 - 2000	0.0	0 - 0	-4.71	<0.0001	***
Adamawa	2021 - date	2009 - 2012	6.2	5 - 7.34	6.17	<0.0001	***
Akwa Ibom	2005 - 2008	1997 - 2000	12.0	8.81 - 15.16	3.6	0.097	ns
Akwa Ibom	2013 - 2016	2005 - 2008	0.1	-0.26	-11.88	0.002	**
Anambra	2005 - 2008	1997 - 2000	30.8	22.48 - 39.13	1.24	0.839	ns
Anambra	2013 - 2016	2005 - 2008	0.0	0 - 0	-30.81	<0.0001	***
Bauchi	1997 - 2000	1993 - 1996	11.9	8.64 - 15.06	-1.46	0.672	ns
Bauchi	2013 - 2016	1997 - 2000	0.0	0 - 0	-11.85	<0.0001	***
Bauchi	2017 - 2020	2013 - 2016	0.0	0 - 0	0	NA	ns
Bayelsa	2013 - 2016	1997 - 2000	0.0	0 - 0	-16.25	0.049	*
Bayelsa	2017 - 2020	2013 - 2016	0.1	-0.2	0.05	0.33	ns
Benue	1997 - 2000	1989 - 1992	31.5	27.21 - 35.73	31.47	NA	ns
Benue	2013 - 2016	1997 - 2000	5.2	-11.09	-26.3	<0.0001	***
Cross River	1997 - 2000	1989 - 1992	10.9	7.88 - 14	-1.76	0.443	ns
Cross River	2005 - 2008	1997 - 2000	41.2	24.71 - 57.74	30.29	0.065	ns
Cross River	2009 - 2012	2005 - 2008	5.3	3.79 - 6.82	-35.92	0.05	*
Cross River	2013 - 2016	2009 - 2012	0.0	NA - NA	-5.3	NA	ns
Cross River	2017 - 2020	2013 - 2016	0.0	NA - NA	0	NA	ns
Delta	2013 - 2016	1997 - 2000	2.0	-4.09	-17.12	<0.0001	***
Delta	2017 - 2020	2013 - 2016	3.1	1.4 - 4.73	1.05	0.44	ns
Ebonyi	2009 - 2012	1997 - 2000	3.4	-8.74	-17.58	<0.0001	***
Ebonyi	2013 - 2016	2009 - 2012	18.3	9.86 - 26.66	14.88	0.006	**
Ebonyi	2017 - 2020	2013 - 2016	0.8	0.18 - 1.49	-17.43	0.001	**
Edo	2009 - 2012	1997 - 2000	34.5	21.62 - 47.33	11.03	0.191	ns
Edo	2013 - 2016	2009 - 2012	0.0	0 - 0	-34.48	0.013	*
Edo	2017 - 2020	2013 - 2016	1.8	0.84 - 2.81	1.83	0.001	***
Ekiti	2009 - 2012	1997 - 2000	2.6	-6.39	-12.99	<0.0001	***
Enugu	1997 - 2000	1993 - 1996	32.9	26.21 - 39.55	4.06	0.47	ns
Enugu	2009 - 2012	1997 - 2000	35.5	23.07 - 47.96	2.64	0.724	ns
Enugu	2013 - 2016	2009 - 2012	23.7	14.63 - 32.68	-11.86	0.169	ns
Enugu	2021 - date	2013 - 2016	37.8	31.2 - 44.45	14.17	0.047	*
FCT	1997 - 2000	1989 - 1992	8.7	4.61 - 12.75	-35.58	NA	ns
FCT	2001 - 2004	1997 - 2000	0.0	0 - 0	-8.68	<0.0001	***
FCT	2013 - 2016	2001 - 2004	0.3	-1.14	0.29	0.343	ns
FCT	2021 - date	2013 - 2016	0.0	0 - 0	-0.29	0.343	ns
Gombe	1997 - 2000	1993 - 1996	16.1	12.6 - 19.56	1.8	0.556	ns
Imo	2013 - 2016	1997 - 2000	0.0	0 - 0	-20.81	<0.0001	***
Jigawa	2013 - 2016	1997 - 2000	0.0	NA - NA	-4.62	NA	ns
Jigawa	2017 - 2020	2013 - 2016	0.0	0 - 0	0	NA	ns
Kaduna	1997 - 2000	1989 - 1992	15.1	12.39 - 17.88	3.71	NA	ns

Kaduna	2005 - 2008	1997 - 2000	0.0	0 - 0	-15.13	<0.0001	***
Kaduna	2009 - 2012	2005 - 2008	19.2	13.97 - 24.46	19.21	<0.0001	***
Kaduna	2013 - 2016	2009 - 2012	2.1	-8.07	-17.16	<0.0001	***
Kaduna	2021 - date	2013 - 2016	9.7	-38.11	7.66	0.473	ns
Kano	2013 - 2016	1997 - 2000	0.0	0 - 0	-13.57	<0.0001	***
Kano	2017 - 2020	2013 - 2016	0.0	0 - 0	0	NA	ns
Kebbi	2001 - 2004	1997 - 2000	4.2	-9.08	-2.78	0.352	ns
Kebbi	2013 - 2016	2001 - 2004	0.0	0 - 0	-4.18	0.213	ns
Kebbi	2017 - 2020	2013 - 2016	0.2	0.06 - 0.43	0.24	0.015	*
Kogi	2005 - 2008	1997 - 2000	26.5	NA - NA	4.63	NA	ns
Kogi	2009 - 2012	2005 - 2008	7.6	3.95 - 11.17	-18.94	NA	ns
Kogi	2013 - 2016	2009 - 2012	0.2	-0.78	-7.36	0.002	**
Kwara	2001 - 2004	1997 - 2000	20.7	19.63 - 21.81	1.59	0.281	ns
Kwara	2005 - 2008	2001 - 2004	34.8	28.5 - 41.07	14.06	<0.0001	***
Kwara	2009 - 2012	2005 - 2008	11.7	-45.87	-23.08	0.281	ns
Kwara	2013 - 2016	2009 - 2012	1.3	-3.69	-10.38	0.538	ns
Lagos	2009 - 2012	1997 - 2000	0.0	NA - NA	-4	NA	ns
Lagos	2013 - 2016	2009 - 2012	0.1	-0.31	0.08	NA	ns
Nasarawa	1997 - 2000	1989 - 1992	22.0	16.8 - 27.17	1.81	0.807	ns
Nasarawa	2009 - 2012	1997 - 2000	0.0	0 - 0	-21.99	<0.0001	***
Nasarawa	2017 - 2020	2009 - 2012	0.0	-0.17	0.04	0.334	ns
Nasarawa	2021 - date	2017 - 2020	31.1	12.66 - 49.54	31.06	0.046	*
Niger	2001 - 2004	1997 - 2000	10.2	8.39 - 12	-3.95	0.001	***
Niger	2009 - 2012	2001 - 2004	0.0	0 - 0	-10.19	<0.0001	***
Niger	2013 - 2016	2009 - 2012	0.0	0 - 0	0	NA	ns
Niger	2017 - 2020	2013 - 2016	5.3	NA - NA	5.33	NA	ns
Niger	2021 - date	2017 - 2020	10.0	NA - NA	4.67	NA	ns
Ogun	2009 - 2012	1997 - 2000	12.5	9.09 - 15.88	-1.73	0.455	ns
Ogun	2013 - 2016	2009 - 2012	2.1	-4.49	-10.43	<0.0001	***
Ogun	2017 - 2020	2013 - 2016	16.9	12 - 21.72	14.81	<0.0001	***
Ogun	2021 - date	2017 - 2020	16.4	11.51 - 21.32	-0.45	0.9	ns
Ondo	2009 - 2012	1997 - 2000	16.6	-35.72	-1.74	0.861	ns
Osun	2001 - 2004	1997 - 2000	30.4	20.85 - 39.98	12.79	0.047	*
Osun	2009 - 2012	2001 - 2004	17.5	10.71 - 24.23	-12.95	0.06	ns
Osun	2013 - 2016	2009 - 2012	15.9	10.36 - 21.51	-1.53	0.739	ns
Oyo	1997 - 2000	1989 - 1992	18.3	16.45 - 20.22	10.33	NA	ns
Oyo	2009 - 2012	1997 - 2000	22.6	16.53 - 28.59	4.23	0.205	ns
Oyo	2013 - 2016	2009 - 2012	4.2	-9.14	-18.36	<0.0001	***
Oyo	2017 - 2020	2013 - 2016	0.1	-0.3	-4.09	0.095	ns
Oyo	2021 - date	2017 - 2020	30.2	21.57 - 38.87	30.11	<0.0001	***
Plateau	1993 - 1996	1989 - 1992	40.0	NA - NA	33.87	NA	ns
Plateau	1997 - 2000	1993 - 1996	19.3	13.37 - 25.28	-20.68	NA	ns
Plateau	2005 - 2008	1997 - 2000	14.5	0.73 - 28.18	-4.87	0.549	ns
Plateau	2009 - 2012	2005 - 2008	0.3	-1.17	-14.16	0.113	ns
Plateau	2017 - 2020	2009 - 2012	0.0	-0.09	-0.27	0.426	ns
Plateau	2021 - date	2017 - 2020	20.0	NA - NA	19.98	NA	ns
Rivers	2013 - 2016	1997 - 2000	0.0	0 - 0	-9.6	0.002	**
Rivers	2017 - 2020	2013 - 2016	0.0	0 - 0	0	NA	ns

Sokoto	2013 - 2016	1997 - 2000	0.0	NA - NA	-1.62	NA	ns
Sokoto	2017 - 2020	2013 - 2016	0.0	0 - 0	0	NA	ns
Taraba	2009 - 2012	1997 - 2000	14.0	4.41 - 23.58	-1.64	0.752	ns
Taraba	2013 - 2016	2009 - 2012	3.3	0.88 - 5.66	-10.73	0.054	ns
Taraba	2021 - date	2013 - 2016	47.4	42.34 - 52.48	44.14	0.013	*
Yobe	2017 - 2020	1997 - 2000	0.0	0 - 0	-12.53	<0.0001	***
Zamfara	2009 - 2012	1997 - 2000	0.0	0 - 0	-5.55	<0.0001	***
Zamfara	2013 - 2016	2009 - 2012	0.0	0 - 0	0	NA	ns
Zamfara	2017 - 2020	2013 - 2016	0.0	0 - 0	0	NA	ns

S4 Appendix Table D: Number and proportion of states within predicted prevalence (%) classes across survey periods.

Period	Prevalence Class	No. of States	Total No. of States (n=37)	Percentage
1997-2000	2-5%	3	37	8.1
1997-2000	5-10%	8	37	21.6
1997-2000	10-30%	24	37	64.9
1997-2000	Above 30%	2	37	5.4
2009-2012	5-10%	16	37	43.2
2009-2012	10-30%	21	37	56.8
2013-2016	0-2%	12	37	32.4
2013-2016	2-5%	15	37	40.5
2013-2016	5-10%	6	37	16.2
2013-2016	10-30%	3	37	8.1
2013-2016	Above 30%	1	37	2.7
2017-2020	0-2%	26	37	70.3
2017-2020	2-5%	9	37	24.3
2017-2020	5-10%	2	37	5.4
2021-present	0-2%	32	37	86.5
2021-present	2-5%	3	37	8.1
2021-present	5-10%	2	37	5.4

S4 Appendix Table E: Number and proportion of southern states within predicted prevalence (%) classes across survey periods.

Period	Prevalence Class	No. of States	Total No. of States	Percentage
1997-2000	5-10%	1	17	5.9
1997-2000	10-30%	14	17	82.4
1997-2000	Above 30%	2	17	11.8
2009-2012	5-10%	5	17	29.4
2009-2012	10-30%	12	17	70.6
2013-2016	0-2%	3	17	17.6
2013-2016	2-5%	7	17	41.2
2013-2016	5-10%	3	17	17.6
2013-2016	10-30%	3	17	17.6
2013-2016	Above 30%	1	17	5.9
2017-2020	0-2%	7	17	41.2
2017-2020	2-5%	8	17	47.1
2017-2020	5-10%	2	17	11.8
2021-present	0-2%	12	17	70.6
2021-present	2-5%	3	17	17.6
2021-present	5-10%	2	17	11.8

S4 Appendix Table F: Linear mixed-effects model results assessing temporal trends in state-level model-predicted prevalence (%) across survey periods, with Holm-adjusted pairwise contrasts

Fixed effect	Estimate	Std. Error	df	t value	p-value
Intercept (1997–2000)	0.1516	0.0072	155.8	21.05	<0.001
2009–2012	−0.0441	0.0091	144	−4.84	<0.001
2013–2016	−0.1036	0.0091	144	−11.36	<0.001
2017–2020	−0.1375	0.0091	144	−15.07	<0.001
2021–present	−0.1414	0.0091	144	−15.50	<0.001

S4 Appendix Table G: Pairwise comparisons of model-predicted prevalence (%) across survey periods.

Comparison	Change (%)	95% CI	p-value
1997–2000 vs 2009–2012	4.41	1.81 to 7.02	<0.001
1997–2000 vs 2013–2016	10.36	7.76 to 12.96	<0.001
1997–2000 vs 2017–2020	13.75	11.15 to 16.35	<0.001
1997–2000 vs 2021–present	14.14	11.54 to 16.75	<0.001
2009–2012 vs 2013–2016	5.95	3.35 to 8.55	<0.001
2009–2012 vs 2017–2020	9.34	6.74 to 11.94	<0.001
2009–2012 vs 2021–present	9.73	7.13 to 12.33	<0.001
2013–2016 vs 2017–2020	3.39	0.79 to 5.99	<0.001
2013–2016 vs 2021–present	3.78	1.18 to 6.38	<0.001
2017–2020 vs 2021–present	0.39	−2.21 to 2.99	0.67

S4 Appendix Table H: Overall effect of survey period model-predicted prevalence from linear mixed-effects model.

Effect	Num df	Den df	F value	p-value
Period	4	144	91.17	<0.001
